# Manipulating Ligand Density at the Surface of Polyoxovanadate-Alkoxide Clusters

**DOI:** 10.1021/acs.inorgchem.3c02314

**Published:** 2023-09-15

**Authors:** Thompson V. Marinho, Eric Schreiber, Rachel E. Garwick, William W. Brennessel, Ellen M. Matson

**Affiliations:** Department of Chemistry, University of Rochester, Rochester, New York 14627, United States

## Abstract

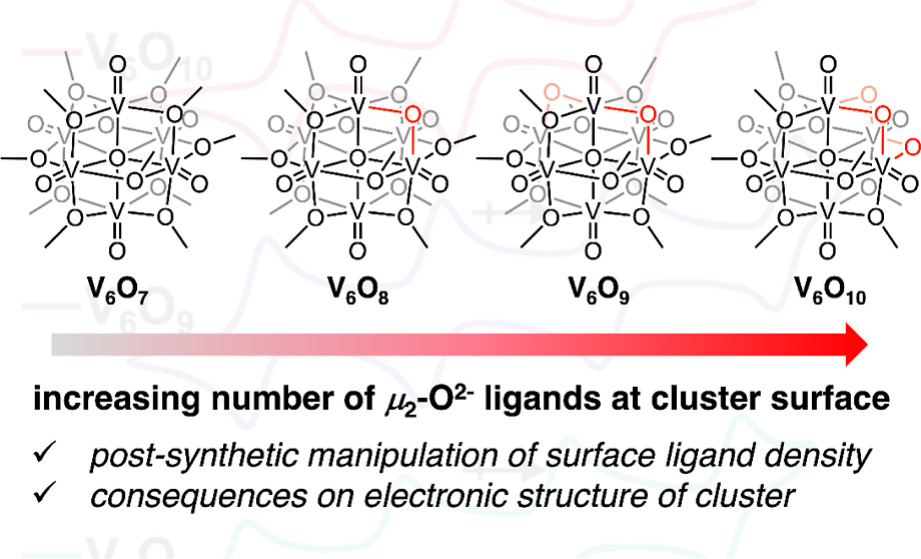

We present the post-synthetic modification of a polyoxovanadate-alkoxide (POV-alkoxide) cluster via the reactivity of its cationic form, [V_6_O_7_(OCH_3_)_12_]^1+^, with water. This result indicates that cluster oxidation increases the lability of bridging methoxide ligands, affording a ligand exchange reaction that serves to compensate for the increased charge of the cluster core. This synthetic advance affords the isolation of a series of POV-alkoxide clusters with varying degrees of μ_2_–O^2–^ ligands incorporated at the surface, namely, [V_6_O_8_(OCH_3_)_11_], [V_6_O_9_(OCH_3_)_10_], and [V_6_O_10_(OCH_3_)_9_]. Characterization of the POV-alkoxide clusters is described; changes in the infrared and electronic absorption spectra are consistent with the oxidation of the cluster core. We also examine the consequences of ligand substitution on the redox properties of the series of POV-alkoxide clusters via cyclic voltammetry; decreased alkoxide ligand density translates to a cathodic shift of analogous redox events. Ligand substitution also increases comproportionation constants of the Lindqvist core, indicating electron exchange between vanadium centers is promoted in structures with greater numbers of μ_2_–O^2–^ ligands.

## Abstract 2

The manipulation of the density of surface alkoxide ligands on Lindqvist-type polyoxovanadate clusters via the reaction of the oxidized form of the assembly with water is reported. The consequences of the removal of bridging alkoxide ligands on the electronic structure of the hexavanadate assembly are described.

## Introduction

Over the past 20 years, metal oxide nanoparticles have been the subject of increased attention due to their relevance in biomedical,^1−4^ sensing,^5^ and energy storage/conversion^6,7^ applications. The growing interest in this subset of nanomaterials lies in their high surface-to-volume ratio, resulting in thermal, electrical, and optical behaviors that are distinct in comparison to those of their bulk congeners.^8^ In almost all applications of metal oxide nanocrystals, an important challenge is the development of design criteria and synthetic strategies for predictable surface functionalization of the metal oxide core with organic ligands. These important chemical modifications improve the solubility of discrete nanoparticles in solution and often dictate the electronic properties and reactivity of the nanoparticle.^8^ However, given the complexity and dynamic nature of nanoparticle surfaces, it remains challenging to gain atomic-level insights into capping ligand density.

Polyoxometalates (POMs) are three-dimensional molecular assemblies composed of multiple transition metal oxyanions connected via bridging oxide (μ_2_–O^2–^) moieties.^9−11^ POMs exist on a continuum between molecular, monometallic, metal-oxo complexes, and bulk transition metal oxide materials. Importantly, these clusters possess delocalized electronic structures and large surface areas that mimic the pertinent properties of metal oxide nanoparticles. With relevance to this work, POMs can be functionalized with organic ligands, resulting in the formation of hybrid architectures. Most often, organofunctionalized POMs are studied as functional building blocks for well-defined, hierarchical nanomaterials.^12,13^ However, an alternative use of these hybrid assemblies is as model systems for better understanding the role of ligands in controlling the electronic structure and reactivity of a nanoscopic material.

**Figure 1:**
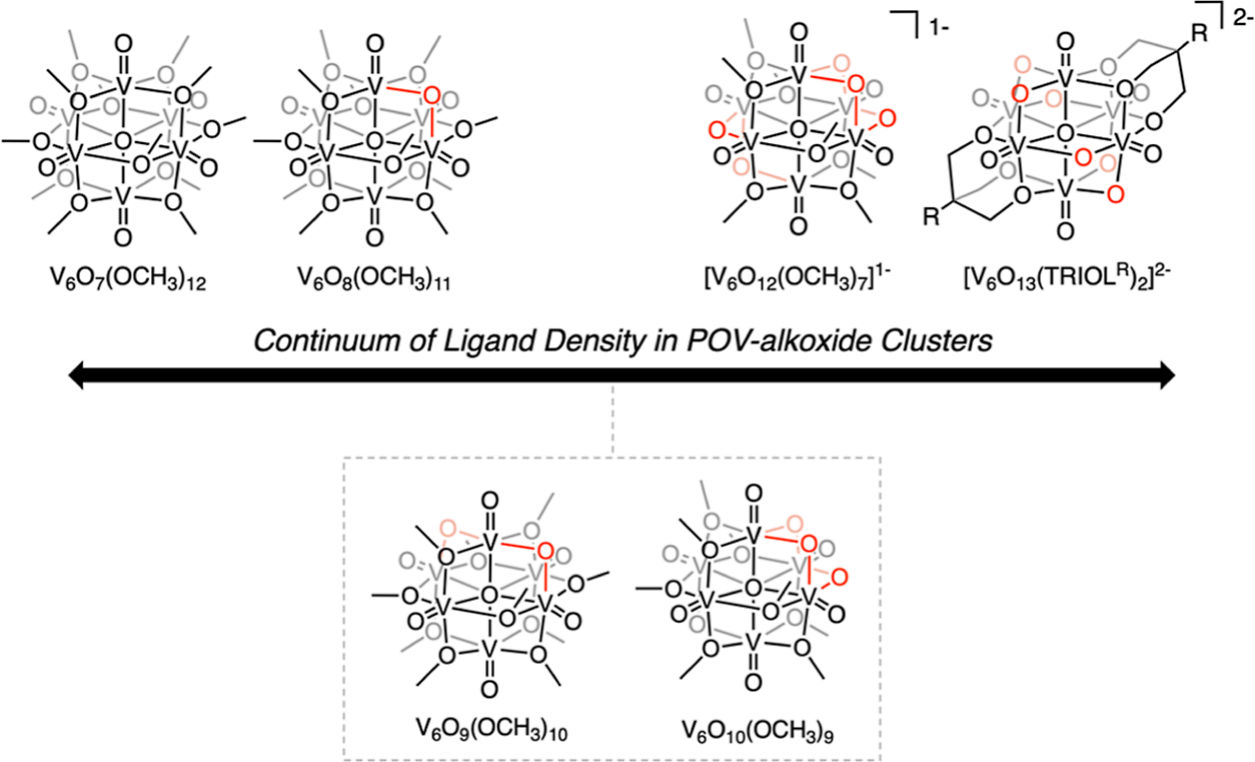
Illustrations of the molecular structures of previously reported POV-alkoxide clusters, demonstrating the diversity of ligand densities established in these organofunctionalized Lindqvist clusters.

Lindqvist-type polyoxovanadate-alkoxide (POV-alkoxide) clusters are a subset of POMs, with diverse surface ligand substitution patterns (Figure 1).^14,15^ Notably, the density of surface ligands can be tailored by modifications to self-assembly synthetic procedures. Early work resulting in the identification of the Lindqvist-type vanadium oxide anion, [V_6_O_13_(TRIOL^CH3^)_2_]^2–^ (TRIOL^CH3^ = (OCH_2_)_3_CCH_3_), was realized by Zubieta and co-workers following addition of excess tris(hydroxymethyl)methane to the decavanadate cluster, [H_3_V_10_O_28_]^3–^.^16^ Incorporation of two tridentate “TRIOL” ligands is essential for cluster formation as the alkoxide moieties serve to mitigate the accumulation of excess negative charge that destabilizes small vanadium oxide assemblies. Subsequently, this synthetic protocol was adapted by Hill and co-workers for the synthesis of a similar POV-alkoxide cluster featuring seven monodentate bridging methoxide ligands, [V_6_O_12_(OCH_3_)_7_]^1–^.^17^ Both examples feature a ∼1:1 ratio of bridging oxide and terminal oxide ligands at the surface of the vanadium oxide core. Over a decade later, a new variant of the POV-alkoxide cluster, [V_6_O_7_(OCH_3_)_12_], was reported by Hartl and co-workers.^18−20^ This cluster features 12 bridging methoxide ligands, occupying all bridging sites at the surface of the assembly.

In the aforementioned studies, the density and identity of organic ligands at the surface of the POV-alkoxide cluster have been demonstrated to directly influence the electronic structure of the vanadium oxide assembly. The examples of POV-alkoxide clusters featuring a mixture of bridging alkoxide and oxide surface ligands are typically isolated in high-valent oxidation states (e.g., ox. state distrib. = V_6_^V^). Electrochemical analysis of [V_6_O_13_(TRIOL^CH3^)_2_]^2–^ and [V_6_O_12_(OCH_3_)_7_]^1–^ reveals two quasi-reversible reduction events for these POV-alkoxide clusters.^16,17^ Zubieta and co-workers have also demonstrated that the *E*_1/2_ value of the sequential reduction processes can be tuned by modifying the electron-donating/withdrawing properties of the tridentate ligands at the surface of the POV-alkoxide cluster, [V_6_O_13_(TRIOL^R^)_2_]^2–^ (R = NO_2_, CH_2_OH, CH_2_N(CH_3_)_2_, and CH_3_).^16^ Our group later elaborated on this work, illustrating that the incorporation of additional bridging methoxide ligands via post-synthetic alkylation shifts the two reduction events toward positive potentials.^21^ In the case of the fully substituted POV-methoxide cluster, V_6_O_7_(OCH_3_)_12_, saturation of the vanadium oxide surface with bridging alkoxide ligands supports access to low-valent electronic configurations for the Lindqvist core. Indeed, in its neutral charge state, the POV-methoxide cluster is isolated as a mixed-valent assembly (ox. state distrib. = V_4_^IV^V_2_^V^), translating to increased conductivity across the Lindqvist ion as a result of intervalence charge transfer.^22^ Electrochemical characterization of V_6_O_7_(OCH_3_)_12_ reveals a rich redox profile, with four quasi-reversible redox events ranging ∼2 V.^18−20,23^ Unlike [V_6_O_13_(TRIOL^R^)_2_]^2–^, the redox properties of the fully substituted Lindqvist core are impervious to changes in electron-donating and electron-withdrawing properties of the surface ligands, indicating the delocalized electronic structure of the reduced assembly is sufficiently decoupled from the inductive effects of the ligands.^24−26^

Recently, our research group described the modified reactivity of a POV-alkoxide cluster with respect to H-atom uptake through the incorporation of a single bridging oxide ligand at the surface of the assembly, [V_6_O_8_(OCH_3_)_11_]^2–^. In the case of [V_6_O_8_(OCH_3_)_11_]^2–^, H-atom transfer occurs exclusively to the nucleophilic bridging oxide position with accelerated reaction rates in comparison to H-atom uptake that takes place at a terminal oxide position in [V_6_O_7_(OCH_3_)_12_]^1–^. Hartl and co-workers originally reported the synthesis of the POV-alkoxide cluster bearing a single bridging oxide ligand, V_6_O_8_(OCH_3_)_11_, as a low-yielding byproduct of the synthesis of V_6_O_7_(OCH_3_)_12_.^20^ Intrigued by the changes in electronic structure and reactivity observed upon modification of surface ligand density in these POV-methoxide clusters, we sought to develop a more reliable synthetic pathway for the formation of V_6_O_8_(OCH_3_)_11_. Herein, we report a new synthetic methodology for post-synthetic modification of ligand density at POV-alkoxide surfaces, leveraging the lability of alkoxide ligands in high oxidation states of the cluster. We also describe the isolation of two new POV-methoxide clusters, V_6_O_9_(OCH_3_)_10_ and V_6_O_10_(OCH_3_)_9_, which feature additional bridging oxide ligands. Collectively, analysis of the electronic structures of the series of POV-alkoxide clusters reveals the consequence of incorporation of bridging oxide ligands in low-valent Lindqvist-type vanadium oxide assemblies, with implications for future studies into the reactivity of these systems.

## Experimental Section

### General Considerations

Unless otherwise noted, all manipulations were carried out in the absence of water and oxygen using standard Schlenk techniques or in a UniLab MBraun inert atmosphere dry box under a dinitrogen atmosphere. All glassware was oven-dried for a minimum of 4 h and cooled in an evacuated antechamber prior to use in the dry box. Solvents were dried and deoxygenated on a glass contour system (Pure Process Technology, LLC) and stored over 3 Å molecular sieves purchased from Fisher Scientific and activated prior to use. NOSbF_6_ and AgOTf were purchased from Sigma-Aldrich and used as received. V_6_O_7_(OCH_3_)_12_ (**V**_**6**_**O**_**7**_), [V_6_O_7_(OCH_3_)_12_]^1–^, [V_6_O_7_(OCH_3_)_12_]^2–^, [V_6_O_7_(OC_2_H_5_)_12_]^1+^, and [V_6_O_7_(OC_2_H_5_)_12_]^2+^ were generated following literature precedent.^20^

^1^H NMR spectra were recorded at 500 MHz on a Bruker DPX-500 spectrometer locked on the signal of deuterated solvents. All chemical shifts were reported relative to the peak of the residual H signal in deuterated solvents. CD_3_CN and CDCl_3_ were purchased from Cambridge Isotope Laboratories, degassed by three freeze–pump–thaw cycles, and stored over fully activated 3 Å molecular sieves. D_2_O was also purchased from Cambridge Isotope Laboratories and degassed by sparging with argon for 1 h prior to use. UV-Vis-NIR spectroscopy was collected using an Agilent Cary 6000i spectrometer at room temperature. Samples were prepared in the dry box in MeCN, added to air-free cuvettes, and sealed prior to removing them from the dry box. All molar absorptivity values were determined by averaging spectra collected in triplicate at different concentrations. Mass spectrometry analyses were performed on an Advion Expression^L^ Compact mass spectrometer equipped with an electrospray probe and an ion-trap mass analyzer (instrument error: ±0.1 amu). Direct injection analysis was employed in all cases with a sample solution in MeCN. Infrared (FT-IR, ATR) spectra of compounds were recorded on a Shimadzu IRAffinity-1 FT-IR spectrophotometer and are reported in wavenumbers (cm^–1^).

Cyclic voltammetry (CV) was performed using a BioLogic SP 150 potentiostat/galvanostat and the EC-Lab software suite. Glassy carbon disks (3 mm, CH Instruments, USA) were used as working electrodes. Working electrodes were polished using a microcloth pad and 0.05 μM alumina powder. Potentials recorded during CV were measured relative to a nonaqueous Ag/Ag^+^ reference electrode with 1 mM AgNO_3_ and 100 mM [^*n*^Bu_4_N]PF_6_ in MeCN (BASi) and ultimately referenced against the Fc^+/0^ couple using an internal reference. A platinum wire served as the counter electrode. All experiments were carried out at room temperature inside a nitrogen-filled glove box (MBraun, USA). All CV measurements were iR compensated at 85% with impedance taken at 100 kHz using the ZIR tool included with the EC-Lab software. CV experiments were conducted at 100 mV/s on solutions of 1 mM analyte and 100 mM [^*n*^Bu_4_N]PF_6_ supporting electrolyte in MeCN.

Single crystals of V_6_O_9_(OCH_3_)_10_ (**V**_**6**_**O**_**9**_^**0**^) and V_6_O_10_(OCH_3_)_9_ (**V**_**6**_**O**_**10**_^**0**^) were mounted on the tip of a thin glass optical fiber (goniometer head) and on an XtaLab Synergy-S Dualflex diffractometer equipped with a HyPix-6000HE HPC area detector for data collection at 99.98(10)−100.00(10) K, respectively. The structures were solved using SHELXT-2018/2^27^ and refined using SHELXL-2018/3^28^ (Table S1). Elemental analyses were performed on a PerkinElmer 2400 Series II analyzer, at the CENTC Elemental Analysis Facility, University of Rochester.

### Synthesis of [V_6_O_7_(OCH_3_)_12_]OTf (**V**_**6**_**O**_**7**_^**1+**^)

The procedure for the synthesis of **V**_**6**_**O**_**7**_^**1+**^ is adapted from previous reports.^23,29^ In the glovebox, a 20 mL scintillation vial was charged with **V**_**6**_**O**_**7**_ (0.560 g, 0.708 mmol), dichloromethane (10 mL), and a stir bar. In a separate 20 mL scintillation vial, a slurry of AgOTf (0.237 g, 0.920 mmol) in dichloromethane (5 mL) was prepared in the dark and added to the POV-alkoxide cluster solution using an excess of solvent. The mixture of oxidant and cluster was stirred for 2 h and subsequently filtered over Celite to remove Ag. The residual solvent was removed under reduced pressure, resulting in the isolation of the product, **V**_**6**_**O**_**7**_^**1+**^, as a green powder. Yield: 0.609 g, 0.648 mmol, 92%. Characterization of **V**_**6**_**O**_**7**_^**1+**^ matches previously reported data.^30^

### Synthesis of V_6_O_8_(OCH_3_)_11_ (**V**_**6**_**O**_**8**_)

The following reaction was run under ambient conditions. A 250 mL round-bottom flask was charged with **V**_**6**_**O**_**7**_^**1+**^ (0.574 g, 0.612 mmol), acetonitrile (100 mL), and a stir bar. Deionized water (1.65 mL, 91.7 mmol) was added dropwise with vigorous stirring. The green solution was stirred for 2 h. Volatile solvents were removed under reduced pressure, leaving a dark green residue. The product was isolated via column chromatography; the reaction mixture was taken up in minimal dichloromethane and chromatographed with hexane/acetone (95:5 by volume) on 230–400 mesh silica gel (SiliaFlash P60). The first green fraction, containing V_6_O_7_(OCH_3_)_12_, was discarded. The second green fraction was collected and evaporated to dryness. The product, **V**_**6**_**O**_**8**_, was dried under high vacuum for 16 h and subsequently brought into the glovebox for analysis. Yield: 0.206 g, 0.266 mmol, 44%. ^1^H NMR (500 MHz, CD_3_CN): δ = 20.44, 19.35, 12.31, 11.20 ppm. FT-IR (ATR, cm^–1^): 1009 (O–CH_3_), 969 (V=O). UV-Vis-NIR (CH_3_CN, 21 °C): 391 nm (ε = 8059 M^–1^ cm^–1^), 687 nm (ε = 461 M^–1^ cm^–1^), 1038 nm (ε = 903 M^–1^ cm^–1^).

### Synthesis of V_6_O_9_(OCH_3_)_10_ (**V**_**6**_**O**_**9**_)

The title compound was isolated as a byproduct during the synthesis of **V**_**6**_**O**_**8**_. During column chromatography, a brown/green band was observed following the elution of the first two green bands as described in the original procedure for the synthesis of **V**_**6**_**O**_**8**_. Solvent polarity was increased to elute the band by using a mixture of 92:8 hexane/acetone. The brown/green fraction was isolated and evaporated to dryness, resulting in the isolation of **V**_**6**_**O**_**9**_ as a brown solid. Yield: 0.090 g, 0.118 mmol, 11%. Crystals suitable for single-crystal X-ray diffraction were grown via slow evaporation of a solution of **V**_**6**_**O**_**9**_ in diethyl ether at −30 °C. ^1^H NMR (500 MHz, CD_3_CN): δ = 25.43, 21.58, 17.31, 14.42, 12.14, 8.80, 8.44, 8.07 ppm. FT-IR (ATR, cm^–1^): 1014 (O–CH_3_), 964 (V=O). UV-Vis-NIR (CH_3_CN, 21 °C): 400 nm (ε = 8283 M^–1^ cm^–1^), 1003 nm (ε = 444 M^–1^ cm^–1^). Elemental analysis: Calcd (%) for C_10_H_30_V_6_O_19_ + 0.75C_4_H_10_O (*M*_W_ = 815.57 g mol^–1^): C, 19.15; H, 4.63. Found (%): C, 19.21; H, 4.32.

### Synthesis of [V_6_O_8_(OCH_3_)_11_]SbF_6_ (**V**_**6**_**O**_**8**_^**1+**^)

In the glovebox, a 50 mL pressure vessel was charged with **V**_**6**_**O**_**8**_ (0.300 g, 0.388 mmol), dichloromethane (20 mL), and a stir bar. Separately, a 20 mL scintillation vial was charged with NOSbF_6_ (0.114 g, 0.427 mmol) and a minimal amount of dichloromethane (∼5 mL); the slurry was transferred to the pressure vessel. We note that the oxidizing agent is minimally soluble in dichloromethane; thus, an additional solvent was used to ensure complete transfer of the reagent to the reaction mixture. Upon complete addition of the oxidant, the pressure vessel was sealed, and the mixture was stirred for 2 h. The reaction mixture was transferred to a 20 mL scintillation vial; residual solvents were subsequently removed under reduced pressure, resulting in isolation of the product [V_6_O_8_(OCH_3_)_11_][SbF_6_] as a green solid. Yield: 0.360 g, 0.356 mmol, 92%. ^1^H NMR (500 MHz, CD_3_CN) δ = 15.40, 12.06, 9.56 ppm. FT-IR (ATR, cm^–1^): 998 (O–CH_3_), 984 (V=O). UV-Vis-NIR (CH_3_CN, 21 °C): 396 nm (ε = 11,000 M^–1^ cm^–1^), 986 nm (ε = 657 M^–1^ cm^–1^). Elemental analysis: Calcd for C_11_H_33_V_6_O_19_SbF_6_ + 0.3C_5_H_12_ (*M*_W_ = 1039.63 g mol^–1^): C, 15.02; H, 3.67; N, 0.00. Found: C, 14.82; H, 3.364; N, 0.190.

### Synthesis of V_6_O_10_(OCH_3_)_9_ (**V**_**6**_**O**_**10**_)

The following reaction was conducted under ambient conditions. A 250 mL round-bottom flask was charged with **V**_**6**_**O**_**8**_^**1+**^ (0.227 g, 0.225 mmol), acetonitrile (100 mL), and a stir bar. With vigorous stirring, deionized water (0.081 mL, 4.5 mmol) was added dropwise to the cluster-containing solution via a glass syringe. The reaction was stirred for 66 h, over which time the solution changed from green to brown. The crude reaction mixture was dried under reduced pressure; the resultant green residue was washed with hot diethyl ether (*T* = ∼ 35 °C) and filtered over a bed of Celite. The portion of the reaction mixture insoluble in diethyl ether was dissolved in dichloromethane and passed over the same Celite filter. Dichloromethane was removed under reduced pressure, resulting in the isolation of **V**_**6**_**O**_**10**_ as a light brown solid. Yield: 0.082 g, 0.110 mmol, 49%. Crystals suitable for single-crystal X-ray diffraction were grown from vapor diffusion from a concentrated solution of the title compound in dichloromethane and pentane. ^1^H NMR (500 MHz, CDCl_3_): δ = 11.14, 6.73 ppm. FT-IR (ATR, cm^–1^): 1002 (O–CH_3_), 969 (V=O). UV–vis/NIR (CH_3_CN, 21 °C): 362 nm (ε = 6870 M^–1^ cm^–1^), 992 nm (ε = 230 M^–1^ cm^–1^). Elemental analysis: Calcd (%) for C_9_H_27_V_6_O_19_ + 0.1C_5_H_12_ (*M*_W_ = 752.16 g mol^–1^): C, 15.17; H, 3.78; N, 0.00. Found (%): C, 15.302; H, 3.504; N, 0.054.

## Results and Discussion

### Reactivity of **V**_**6**_**O**_**7**_^**1+**^ with Water

**Figure 2:**
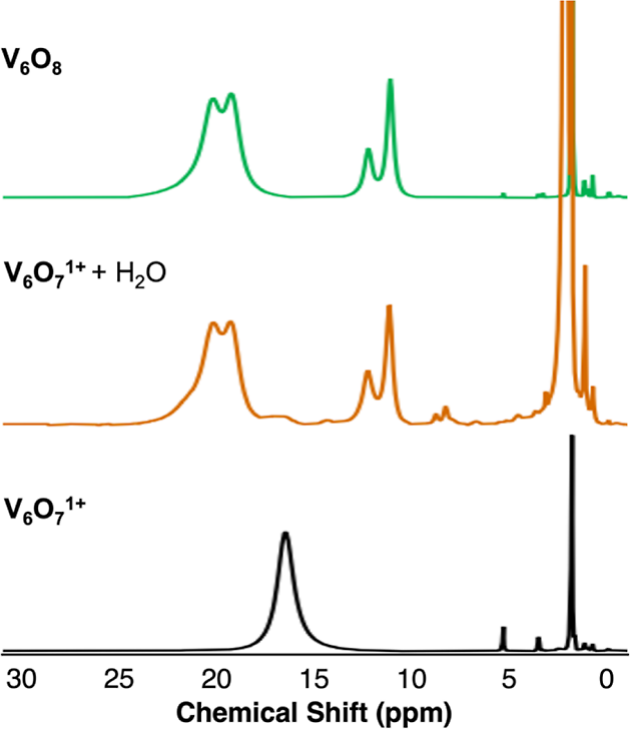
^1^H NMR spectra of [V_6_O_7_(OCH_3_)_12_]OTf (**V**_**6**_**O**_**7**_^**1+**^) before (black, bottom) and after (orange, middle) addition of H_2_O and **V**_**6**_**O**_**8**_ after isolation (green, top) in CD_3_CN at 21 °C.

**Scheme 1:**
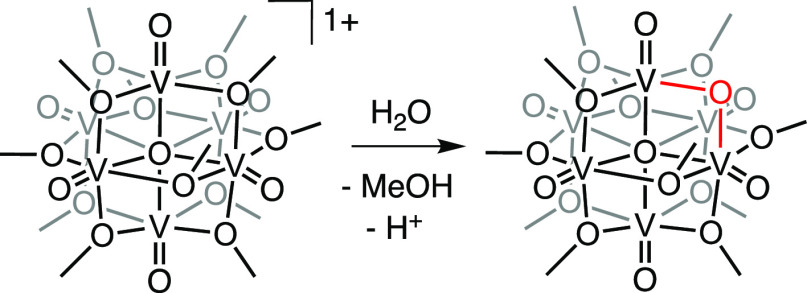
Synthesis of V_6_O_8_(OMe)_11_ (**V**_**6**_**O**_**8**_)

Our interest in the development of a systematic approach for manipulating the density of alkoxide ligands at POV surfaces was born out of a serendipitous discovery. Under the auspices of a separate project focused on understanding oxidatively induced decomposition pathways of POV-alkoxide clusters with relevance to their application in nonaqueous redox flow batteries, our team was investigating the reactivity of the cationic form of the fully substituted POV-methoxide cluster with water. Monitoring the addition of an excess of water to a sample of [V_6_O_7_(OCH_3_)_12_]OTf (**V**_**6**_**O**_**7**_^**1+**^) dissolved in CD_3_CN by ^1^H NMR spectroscopy reveals an immediate reaction (Scheme 1 and Figure 2). The starting material has a single resonance at 16.69 ppm, consistent with the pseudo-octahedral geometry of the fully substituted POV-alkoxide core; all 12 methoxide ligands are positioned in an identical chemical environment, resulting in a single paramagnetically shifted and broadened resonance for this assembly. Upon addition of water, an immediate progression to a spectrum with a pattern of four signals is observed (δ = 20.44, 19.35, 12.31, and 11.20 ppm). This change in distribution of resonances in the ^1^H NMR spectrum of the cluster is consistent with a reduction in the symmetry of the assembly, suggesting a change in surface ligand occupancy. Methanol is also observed in the reaction mixture (Figure S1).

Fortuitously, the pattern of resonances observed by ^1^H NMR spectroscopy was familiar to our team. Previously, our group studied the reactivity of a variant of the POV-alkoxide cluster possessing a single bridging oxide unit, V_6_O_8_(OCH_3_)_11_ (**V**_**6**_**O**_**8**_). The original synthesis of this compound was reported by Hartl and co-workers in 2009; its isolation is possible in a low yield (31%) from the solvothermal reaction of VO(O^*t*^Bu)_3_ in methanol.^20^ The authors note that the formation of **V_6_O_8_** is completely suppressed in the presence of a reductant (e.g., TBABH_4_), suggesting that substitution of the bridging methoxide moiety for an oxido substituent occurs to alleviate the excess positive charge of the hexavanadate assembly. Consistent with this hypothesis, we observe that low charge states of the POV-methoxide cluster do not react with similar amounts of water at similar time points (Figures S2–S4); conversion to **V**_**6**_**O**_**8**_ was only noted after a week of exposure of the neutral POV-methoxide cluster, **V**_**6**_**O**_**7**_, to 150 equiv of water. This result indicates that high charge states of the assembly increase the lability of bridging alkoxide ligands.

Analysis of the crude reaction mixture formed following addition of water to **V**_**6**_**O**_**7**_^**1+**^ by ^1^H NMR spectroscopy suggests that the formation of **V**_**6**_**O**_**8**_ occurs selectively and in high yield. However, further characterization of the crude product via electrospray ionization mass spectrometry (ESI–MS) indicates that a small fraction of POV-alkoxide clusters with higher numbers of bridging oxide ligands are also generated via this preparatory pathway (i.e., V_6_O_19–*x*_(OCH_3_)_*x*_, *x* < 11, Figure S5). This observation is supported by CV of the crude sample (Figure S6). Two sets of semi-overlapping electrochemical waves are observed, with the major species featuring redox potentials at +0.93, +0.36, −0.15, and −0.71 V vs Fc^+/0^ and another set of signals at more positive potentials (+1.1, +0.51, −0.0, and −0.61 V vs Fc^+/0^). The half-wave potentials corresponding to the major product in solution are consistent with values reported previously for **V**_**6**_**O**_**8**_. The additional redox waves appear to correspond to a single compound. This is supported by the similar magnitude in current response across the four events, indicating a consistent concentration of the corresponding redox-active species. In addition, the voltage separation, or Δ*E*_1/2_, between consecutive events (∼0.5 V) is similar in magnitude to that of other known POV-alkoxides (i.e., **V**_**6**_**O**_**7**_ and **V**_**6**_**O**_**8**_), suggesting that the impurity is a hexavanadate cluster.^19,20^ We note that the *E*_1/2_ values of the redox events of the impurity species are anodically shifted from those of **V**_**6**_**O**_**8**_. Given that the redox events of **V**_**6**_**O**_**8**_ are similarly shifted with respect to those of **V**_**6**_**O**_**7**_, we hypothesized that the minor product formed in the reaction of **V**_**6**_**O**_**7**_^**1+**^ with water is the Lindqvist POV-alkoxide featuring an additional μ_2_–O^2–^ moiety, [V_6_O_9_(OCH_3_)_10_] (**V**_**6**_**O**_**9**_, vide infra).

**Figure 3:**
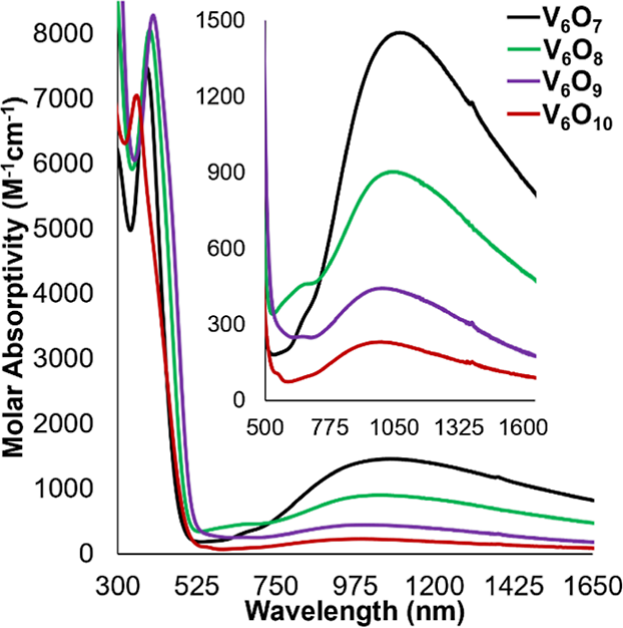
Electronic absorption spectra of **V**_**6**_**O**_**7**_ (black), **V**_**6**_**O**_**8**_ (green), **V**_**6**_**O**_**9**_ (purple), and **V**_**6**_**O**_**10**_ (red) measured in acetonitrile at 21 °C. Inset highlights the near-IR region of the spectrum to illustrate more clearly intervalence charge transfer events of the mixed-valent POV-alkoxide clusters.

Access to an analytically pure sample of **V**_**6**_**O**_**8**_ is possible via column chromatography. The spectroscopic characterization of this complex has been previously reported but is summarized here.^20^ The electronic absorption spectrum of **V**_**6**_**O**_**8**_ features three prominent transitions: an intense band at 391 nm (ε = 8059 M^–1-^ cm^–1^) corresponding to an O_t_ to V^V^ ligand-to-metal charge transfer (LMCT), and two additional weaker broad bands located at 687 nm (ε = 461 M^–1^ cm^–1^) and 1038 nm (ε = 903 M^–1^ cm^–1^) indicative of V^IV^/V^V^ intervalence charge transfer (IVCT) between V^IV^ and V^V^ ions of the core (Figure 3). These transitions are similar to those reported for **V**_**6**_**O**_**7**_ (λ = 382, 1075 nm, and ε = 7475 and 1453 M^–1^ cm^–1^, respectively). The additional feature at 687 nm is assigned to charge transfer across the mixed-valent [V_2_O_3_]^3+^ moiety, resulting from installation of a bridging oxide moiety.^20^ Finally, the IR spectrum of **V**_**6**_**O**_**8**_ features four bands of interest at 1009, 969, 705, and 583 cm^–1^, corresponding to the O–CH_3_ and V=O_t_ stretching frequencies and the V–O_b_–V and V–O_b_(CH_3_)–V bending modes, respectively (Figure S7).

The distribution of cluster complexes formed in the reaction of [V_6_O_7_(OCH_3_)_12_]^1+^ with water suggests that the isolation of more oxidized Lindqvist structures is possible. Indeed, during the isolation of **V**_**6**_**O**_**8**_ by column chromatography, we observed an additional brown band eluting behind the green band containing **V**_**6**_**O**_**8**_. Increasing the polarity of the eluent affords isolation of the brown product; ESI–MS indicates a cluster containing two bridging oxide ligands, **V**_**6**_**O**_**9**_, is present as the sole species in the sample (*m*/*z* 759.8; Figure S8). Characterization of the product via ^1^H NMR spectroscopy reveals a range of signals, with a major distribution of peaks at 17.31, 14.42, 12.14, 8.80, and 8.44 ppm, alongside a series of smaller signals (26–11 and 8 ppm; Figure S9). The quantity of **V**_**6**_**O**_**9**_ isolated by this method is low, yielding only 11% of an analytically pure compound from the reaction mixture; this observation is consistent with the ^1^H NMR of the crude reaction mixture showing **V**_**6**_**O**_**8**_ as the major product.

**Figure 4:**
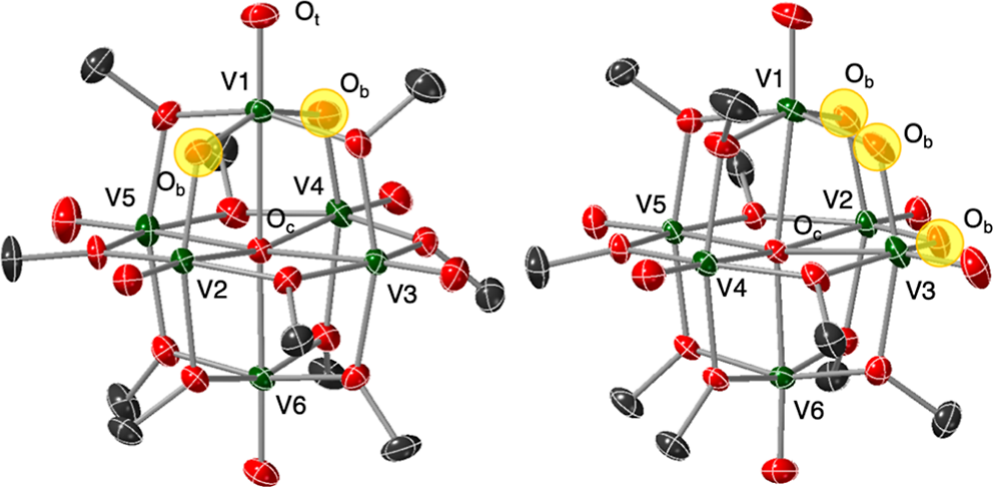
Molecular structures of **V**_**6**_**O**_**9**_ and **V**_**6**_**O**_**10**_ shown with 50% probability ellipsoids. H-atoms omitted for clarity. Key: green = V, red = O, and gray = C. Bridging oxide ligands incorporated at the surface of the assembly are highlighted with yellow spheres for ease of identification.

Single crystals of **V**_**6**_**O**_**9**_ suitable for X-ray diffraction were grown from slow evaporation of a solution of the complex in diethyl ether. Refinement of the data reveals the product is indeed a Lindqvist POV-alkoxide cluster featuring two bridging oxide ligands. Notably, both oxide moieties in the crystallized sample are bound to the same vanadium center in a trans configuration (Figure 4, Table 1, and Table S1 for complete crystallographic parameters). The V–O_b_ bond lengths defining interactions between the μ_2_–O^2–^ ligand and adjacent vanadium centers range from 1.776(5) to 1.933(5) Å. We note the μ_2_–O^2–^ ligands in **V**_**6**_**O**_**9**_ direct oxidation state distributions of vanadium ions across the cluster core; all vanadium centers (V1, V2, and V4) bound to the μ_2_–O^2–^ are found in the 5+ oxidation state [see discussion of bond valence sum (BVS) calculations summarized below]. These values are consistent with distances reported for other Lindqvist-type POV clusters featuring isovalent [V_2_O_3_]^4+^ subunits (V^V^–O_b_–V^V^ = 1.773(4)–2.017(2) Å).^16,17,31−37^ Despite overall retention of the Lindqvist structure, analysis of internal bond lengths and angles obtained for this complex reveals significant core rearrangement upon bridging oxide ligand exchange. For instance, the presence of two bridging oxides about V1 compensates for the positive charge on the metal ion, weakening its interaction with the central oxygen atom, resulting in a significantly longer V1–O_c_ bond distance (2.357(4) Å) in comparison to the average V–O_c_ distance in the molecule (V–O_c_ avg. = 2.269(67) Å). As a result, the distance between the central oxygen atom and the vanadium ion positioned directly across the cluster is shortened (V6–O_c_ = 2.144(4) Å). Additionally, the angles between V centers across O_c_ deviate significantly from the linearity observed for the homoleptic methoxide functionalized complex, **V**_**6**_**O**_**7**_ 174.6(4.1)° vs 178.81(47)°, respectively). The large standard deviation in the internal angles of **V**_**6**_**O**_**9**_ is due in large part to the contraction of V–O_b_ bonds at V2 and V4, pulling these centers out of the molecule’s equatorial plane and producing a contracted V2–O_c_–V4 angle of 168.9(2)°. A similar phenomenon is observed for the V3–O_c_–V5 angle 176.2(2) °C), albeit to a lesser extent as the metal centers remain in the equatorial plane, with O_c_ pushed toward V6. The solid-state structure of **V**_**6**_**O**_**9**_ reveals how rearrangement of the entire core is required to accommodate changes in ligand density.

**Table 1:** Selected Bond Lengths and Angles for **V**_**6**_**O**_**7**_,^19^**V**_**6**_**O**_**9**_, and **V**_**6**_**O**_**10**_^a^

bond	V_6_O_7_	V_6_O_9_	V_6_O_10_
V=O_t_^avg.^	1.592(6) Å	1.592(52) Å	1.592(3) Å
V–O_b_(CH_3_)^avg.^	1.973(40) Å	1.973(45) Å	1.979(55) Å
V–O_b_^avg.^		1.836(60) Å	1.821(58) Å
V_(sat.)_–O_c_^avg.^	2.291(33) Å	2.246(74) Å, (V6 = 2.144(4) Å)	2.161(28) Å
V_(μ2-O)_–O_c_^avg.^		2.292(50) Å, (V1 = 2.357(4) Å)	2.369(26) Å
V–O_c_–V^avg.^	178.81(47)°	174.56(4.14)°	170.99(15)°

a Crystal structure of **V**_**6**_**O**_**8**_ has been described previously; however, the bond parameters are unable to be used in comparative analysis as the cluster crystallized on an inversion center, inhibiting crystallographic identification of the μ_2_–O^2–^ moiety.

No counterions are observed in the structure of **V**_**6**_**O**_**9**_, suggesting the vanadium oxide assembly is isolated in its neutral charge state. A neutral overall charge for **V**_**6**_**O**_**9**_ would necessitate an oxidation state distribution of metal centers of V_2_^IV^V_4_^V^. Because all six V centers crystallize on general positions in the unit cell, we were able to probe oxidation state assignments using BVS calculations (Table S2). BVS analysis confirms the proposed oxidation state distribution of vanadium centers. V–O bond distances surrounding the three vanadium ions bound to μ_2_–O^2–^ ligands (V1, V2, and V4) indicate that these metal centers are in the V^V^ state. In contrast, BVS calculations for V3 and V5, the vanadium centers bound exclusively to bridging alkoxide ligands, indicate the metal centers are in the V^IV^ oxidation state. Finally, V6 was determined in BVS calculations to be a V^V^ center, possibly due to the strong interaction between this metal center and the μ_6_–O^2–^ moiety (vide supra). It is important to note that these oxidation state assignments of individual vanadium centers are made on a solid-state structure at low temperatures, which may serve to localize charges at each V center. At room temperature and in solution, electrons are considered to be substantially delocalized across mixed-valent vanadium centers in the POV-alkoxide cluster, complicating the assignment of formal oxidation states.

The *C*_2*v*_ symmetry observed in the X-ray crystal structure obtained for **V**_**6**_**O**_**9**_ provides structural context for the observed ^1^H NMR spectrum of this compound. The *C*_2_ axis generates five inequivalent methoxide environments across the core with expected integrals in a 1:1:1:1:2 ratio. This is consistent with the largest 5 signals in the spectrum, found at 17.31, 14.42, 12.14, 8.80, and 8.44 ppm. The remaining ^1^H NMR signals could correspond to a different isomer which features another orientation of the μ_2_–O^2–^ moieties and lower symmetry; however, no other isomers were able to be crystallized or observed in the crystallographic data collected for **V**_**6**_**O**_**9**_.

The electronic absorption spectrum of **V**_**6**_**O**_**9**_ is broadly similar to those measured for complexes **V**_**6**_**O**_**7**_ and **V**_**6**_**O**_**8**_. Two absorption features are observed at 400 nm (ε = 8283 M^–1^ cm^–1^) and 1003 nm (ε = 444 M^–1^ cm^–1^), corresponding to LMCT (V^V^=O_t_) and IVCT (V^IV/V^), respectively (Figure 3). Analysis of complex **V**_**6**_**O**_**9**_ via IR spectroscopy reveals transitions at 964, 1014, 757, and 587 cm^–1^, similar to values reported for **V**_**6**_**O**_**8**_ (Figure S7). An additional band at 683 cm^–1^ was observed, attributed to the additional V–O_b_–V bending mode available to **V**_**6**_**O**_**9**_ as a result of the additional bridging oxide ligand.

The observed reactivity of the oxidized form of Hartl’s POV-alkoxide clusters with water has significant implications in molecular design for catalysis. The substitution of a bridging methoxide ligand for a bridging oxide presents a new approach for post-synthetic manipulation of the cluster surface, exposing a nucleophilic site at the periphery of the vanadium oxide assembly. Most notably, this result refutes prior work describing V_6_O_7_(OCH_3_)_12_ as a catalyst for water oxidation.^38^ The reactivity of **V**_**6**_**O**_**7**_^**1+**^ detailed here indicates that before accessing the oxidizing potentials required to oxidize water, the cluster reacts through ligand rearrangement reactions, converting the fully substituted assembly to more oxidized clusters featuring bridging oxide ligands. Current investigations underway in our laboratory are probing the reactivity of the more oxidized variants of POV-alkoxide clusters as electrocatalysts for oxidative transformations.

With respect to our own work focused on the development of charge carriers for nonaqueous redox flow batteries, the reactivity of the oxidized form of the POV-alkoxide cluster with water indicates a point of weakness relevant to this application. The elimination of adventitious water is a persistent challenge at the bulk scale; the observed reactivity at the cluster surface in its oxidized forms presents a deactivation pathway for the charge carrier cycling through positively charged oxidation states in the posolyte chamber. Generally speaking, the instability of **V**_**6**_**O**_**7**_ in its oxidized forms is consistent with prior observations from our group; bulk electrolysis experiments designed to access the +2 charge state of the POV-alkoxide cluster result in complete degradation of the sample.^23^ However, given the anhydrous operating conditions of our original report, **V**_**6**_**O**_**7**_^**1+**^ was deemed sufficiently stable over long periods of time. This finding indicates additional concerns associated with reactivity pathways operating in the presence of small amounts of water.

Prior work from our laboratory has suggested that POV-alkoxide clusters with longer primary alkoxide ligands possess increased stability in their oxidized forms.^23,39,40^ We have previously stated that this observation is due to a change in the inductive effect of long-chain alkoxide surface ligands on the vanadium oxide core. However, an alternative explanation could be associated with a lack of reactivity of POV-alkoxide clusters bearing long-chain surface ligands with water, in which case the aliphatic groups serve to kinetically inhibit the interaction of the oxidized core with the substrate. Indeed, addition of 150 equiv of water to [V_6_O_7_(OC_2_H_5_)_12_]^1+^ results in no reaction, even under prolonged exposure and elevated temperatures (Figure S10). Interestingly, while no ligand loss is observed upon addition of water to the more oxidized form of the POV-ethoxide, [V_6_O_7_(OC_2_H_5_)_12_]^2+^, the cluster is reduced by one electron in the presence of water (Figure S11); this preliminary result is beyond the scope of the current study but is under active investigation by our research team.

### Synthesis of Further Oxidized Forms of POV-Alkoxide Clusters

**Scheme 2:**
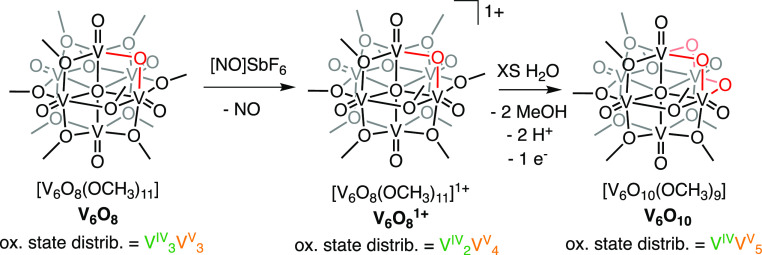
Syntheses of **V**_**6**_**O**_**8**_^**1+**^ and **V**_**6**_**O**_**10**_

The isolation of **V**_**6**_**O**_**9**_ reveals that the Lindqvist core structure of monodentate alkoxide-bridged POV clusters can accommodate bridging oxide exchange; however, this comes with significant alterations to the electronic structure of the complex. Interested in further determining trends in cluster properties upon exchange of bridging alkoxide moieties, we set out to generate clusters with decreased alkoxide densities. Toward this goal, we hypothesized that the chemical oxidation of **V**_**6**_**O**_**8**_ would provide access to an appropriate starting material for subsequent ligand exchange. Exposure of **V**_**6**_**O**_**8**_ to 1 equiv of nitrosonium hexafluoroantimonate (NOSbF_6_) in DCM produces the 1e^–^ oxidized complex, [V_6_O_8_(OCH_3_)_11_][SbF_6_] (**V**_**6**_**O**_**8**_^**1+**^) in excellent yield (92%; Scheme 2). The ^1^H NMR spectrum of **V**_**6**_**O**_**8**_^**1+**^ reveals three paramagnetically shifted and broadened resonances corresponding to methoxide protons decorating the surface of the assembly (Figure S12). The formation of the desired product was confirmed by elemental analysis (see the Experimental Section for details).

Additional characterization of **V**_**6**_**O**_**8**_^**1+**^ was performed by electronic absorption and IR spectroscopy. The electronic absorption spectrum of **V**_**6**_**O**_**8**_^**1+**^ features two absorption bands located at 396 nm (ε = 11,000 M^–1^ cm^–1^) and 986 nm (ε = 660 M^–1^ cm^–1^). The decrease in energy and increase in molar absorptivity of the V^V^=O_t_ LMCT band relative to the neutral redoxomer are consequences of the increased population of V^V^ centers within the Lindqvist core. Similarly, the increase in energy and decrease in molar absorptivity of the IVCT band are due to the presence of only two V^IV^ centers, decreasing the probability of charge transfer between mixed-valent vanadium centers. In the IR spectrum of **V**_**6**_**O**_**8**_^**1+**^, the V=O_t_ vibration is observed at 986 cm^–1^, shifted to a higher frequency in comparison to the analogous transition of **V**_**6**_**O**_**8**_ as a consequence of decreased V=O_t_ bond lengths upon oxidation (**V**_**6**_**O**_**8**_ = 1.591 Å, ν(V=O_t_) = 969 cm^–1^ and **V**_**6**_**O**_**8**_^**1+**^ = 1.581 Å, ν(V=O_t_) = 986 cm^–1^). Cluster oxidation does not have a substantial effect on the positions of the remaining vibrational bands (Figure S13).

With **V**_**6**_**O**_**8**_^**1+**^ in hand, our attention turned to its reactivity with water in MeCN. Addition of 20 equiv of water to **V**_**6**_**O**_**8**_^**1+**^ results in a gradual color change from green to brown. Analysis of the crude reaction mixture via ESI–MS after 66 h reveals complete consumption of the starting material. To our surprise, the major product was not **V**_**6**_**O**_**9**_; instead, a peak was observed at *m*/*z* = 745, consistent with the formation of V_6_O_10_(OCH_3_)_9_ (**V**_**6**_**O**_**10**_; Figure S14).

Unambiguous determination of the identity of the product was performed by X-ray crystallography. Following a brief work-up (see Experimental Section for additional details), single crystals suitable for analysis of **V**_**6**_**O**_**10**_ were grown from vapor diffusion of pentane into a concentrated solution of the product in DCM. Refinement of the data revealed the expected product, **V**_**6**_**O**_**10**_, which features three bridging oxide ligands positioned at a single face of the cluster (Figure 4, Table 1, and Table S1 for complete crystallographic parameters). This arrangement is surprising given the fact that the crystal structure of **V**_**6**_**O**_**9**_ possesses bridging oxide ligands positioned on opposite faces based on crystallographic data. This result suggests that either a mixture of products can be formed or alternatively formation of **V**_**6**_**O**_**10**_ occurs without proceeding through **V**_**6**_**O**_**9**_. That being said, the bond metrics of **V**_**6**_**O**_**10**_ generally resemble those observed for **V**_**6**_**O**_**9**_; the central μ_6_–O^2–^ ligand distorts away from the vanadium centers bound to surface μ_2_–O^2–^ ligands, with bond distances ranging from 2.3325(19) to 2.390(2) Å. Two of these distances are slightly longer than the V1–O_c_ distance observed in **V**_**6**_**O**_**9**_ (2.357(4) Å; note that V1 is the vanadium ion in **V**_**6**_**O**_**9**_ coordinated to two μ_2_–O^2–^ ligands), consistent with the increased density of μ_2_–O^2–^ ligands, and thus charge on a single face of **V**_**6**_**O**_**10**_. The V–O_b_ distances range from 1.762(3) to 1.889(2) Å; the shortest V–O_b_ distances in **V**_**6**_**O**_**10**_ are shorter than those of **V**_**6**_**O**_**9**_; however, the average V–O_b_ distances remain constant between the two assemblies. BVS calculations were performed on **V**_**6**_**O**_**10**_ to probe oxidation state assignments of the Lindqvist core (Table S3). This analysis predicts a mixed-valent electron structure of **V**_**6**_**O**_**10**_ (V^IV^V_5_^V^), consistent with the anticipated distribution of oxidation states hypothesized based on charge balancing calculations.

Complex **V**_**6**_**O**_**10**_ was further characterized by electronic absorption and IR spectroscopy. Like the other mixed-valent POV-alkoxide clusters described in this work, **V**_**6**_**O**_**10**_ possesses two diagnostic transitions observed at 362 nm (ε = 6879 M^–1^ cm^–1^) and 992 nm (ε = 230 M^–1^ cm^–1^), corresponding to LMCT across V^V^=O_t_ and V^IV/V^ IVCT, respectively (Figure 3). The intervalence charge transfer band is significantly weaker than those of **V**_**6**_**O**_**7**_, **V**_**6**_**O**_**8**_, and **V**_**6**_**O**_**9**_, consistent with the reduced number of V^IV^ ions contained within the hexavanadate core of **V**_**6**_**O**_**10**_. The IR spectrum of **V**_**6**_**O**_**10**_ contains transitions at 1002 and 960 cm^–1^, assigned to the O–CH_3_ and V=O_t_ stretching modes, respectively (Figure S7). These values closely resemble those reported for **V**_**6**_**O**_**9**_ (vide supra).

### Electrochemical Characterization of V_6_O_*n*_ (*n* = 7, 8, 9, and 10)

The combination of multiple redox-active metal centers within a single molecule imparts POMs with rich electrochemical profiles. This unique trait renders POMs attractive candidates for applications in electrocatalysis and electrochemical energy storage.^41−43^ To this end, the study of POMs’ electrochemical properties is critical in the development of structure–property relationships, providing design criteria for these clusters.

With relevance to this report, our group is interested in modifying the molecular composition of POV-alkoxide clusters to tune their electrochemical profiles. For example, the installation of substitutional cationic (i.e., heterometals)^44−48^ and anionic dopants (i.e., O-atom defects, halides)^49−51^ into POV-alkoxides has been shown to shift the redox profiles of these complexes anodically and cathodically, respectively. In addition, the redox properties of these POV-alkoxides are sensitive to the surrounding electrolyte, with the presence of coordinating cations (i.e., alkali ions and protons) facilitating cluster reduction at more modest potentials and allowing a greater number of reducing equivalents to be injected into a given molecule.^52−54^ Finally, the incorporation of surface functionalities has been employed to alter the charge transfer characteristics of POMs, with surface ligand electronics and density both serving as synthetic handles to elicit changes in electrochemical properties.^16,20,21^

**Figure 5:**
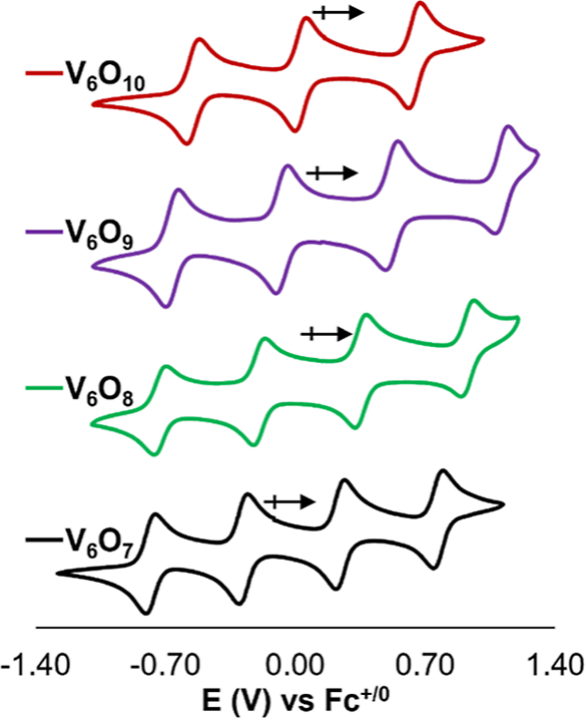
CVs of **V**_**6**_**O**_**7**_ (black), **V**_**6**_**O**_**8**_ (green), **V**_**6**_**O**_**9**_ (purple), and **V**_**6**_**O**_**10**_ (red) measured in acetonitrile with 0.1 M [^*n*^Bu_4_N]PF_6_ as the supporting electrolyte. Voltammetric data collected at a scan rate of 100 mV/s.

To investigate the influence of μ_2_–O^2–^ moieties on the redox properties of this family of POV-alkoxide clusters, we performed electrochemical characterization of the series of complexes reported in this work. The CVs of **V**_**6**_**O**_**7**_ and **V**_**6**_**O**_**8**_ have been reported elsewhere, but we summarize Hartl’s discussion here as it is pertinent to the development of a full picture of the consequences of ligand substitution.^20^ The electrochemical profiles of **V**_**6**_**O**_**7**_ and **V**_**6**_**O**_**8**_ feature four quasi-reversible redox events spanning a 2 V window in MeCN (Figure 5 and Table 2). Notably, each electrochemical wave corresponds to a 1e^–^ redox process; in the oxidative sweep, this electron transfer event can be conceptualized by the conversion of a single vanadium(IV) center to vanadium(V). In reality, charge is delocalized across the cluster core, resulting in the removal of a single electron from a “cloud” of electron density distributed across the Lindqvist ion. The most reduced forms of **V**_**6**_**O**_**7**_ and **V**_**6**_**O**_**8**_ possess oxidation state distributions of V_6_^IV^ and V_5_^IV^V^V^, respectively.^55^ As such, when directly compared to analogous redox couples in **V**_**6**_**O**_**7**_, each event of **V**_**6**_**O**_**8**_ is cathodically shifted (Δ*E*_1/2,avg_ = −0.44 V), indicating that substitution of a bridging methoxide ligand by a bridging oxide moiety stabilizes oxidized forms of the hexavanadate assembly (Table 2).

**Table 2:** Electrochemical Parameters Measured for **V**_**6**_**O**_**7**_, **V**_**6**_**O**_**8**_, **V**_**6**_**O**_**9**_, and **V**_**6**_**O**_**10**_ Measured in MeCN with 0.1 M [^n^Bu_4_N]PF_6_ as the Supporting Electrolyte (100 mV/s)^a^

redox event	V_6_O_7_(OMe)_12_ (V)	V_6_O_8_(OMe)_11_ (V)	V_6_O_9_(OMe)_10_ (V)	V_6_O_10_(OCH_3_)_9_ (V)
V_5_^IV^V^V^ + e^–^ ⇌ V_6_^IV^	–0.78			
V_4_^IV^V_2_^V^ + e^–^ ⇌ V_5_^IV^V^V^	–0.28	–0.71		
V_3_^IV^V_3_^V^ + e^–^ ⇌ V_4_^IV^V_2_^V^	+0.25	–0.15	–0.67	
V_2_^IV^V_4_^V^ + e^–^ ⇌ V_3_^IV^V_3_^V^	+0.79	+0.36	–0.07	–0.55
V^IV^V_5_^V^ + e^–^ ⇌ V_2_^IV^V_4_^V^		+0.93	+0.52	+0.03
V_6_^V^ + e^–^ ⇌ V^IV^V_5_^V^			+1.10	+0.64

a All redox potentials are referenced vs Fc^+/0^.

Next, we analyzed the CV of **V**_**6**_**O**_**9**_ (Figure 5 and Table 2). Four quasi-reversible redox processes were observed with *E*_1/2_ values of +1.10, +0.52, −0.07, and −0.67 V (vs Fc^+/0^); it is worth noting that the electrochemical profile is consistent with the additional redox events observed in the crude reaction mixture of **V**_**6**_**O**_**7**_^**1+**^ and water (Figure S6; vide supra). The neutral cluster possesses an open circuit voltage of 0.14 V (vs Fc^+/0^), indicating that the cluster can be reduced by 2 electrons from the neutral state. Based on the oxidation state assignment of **V**_**6**_**O**_**9**_ provided by BVS calculations, we hypothesize that the most reduced form of the cluster would possess an oxidation state distribution of V_4_^IV^V_2_^V^. This finding suggests that the exchange of bridging alkoxides for μ_2_–O^2–^ ligands restricts the ability of the cluster to take on reducing equivalents. Finally, despite the apparent anodic shift in the CV of **V**_**6**_**O**_**9**_ vs **V**_**6**_**O**_**8**_, a comparison of analogous redox couples (i.e., V_2_^IV^V_4_^V^ + e^–^ ⇌ V_3_^IV^V_3_^V^) reveals a cathodic shift (Δ*E*_1/2,avg_ = −0.44 V) in the electrochemical profile upon installation of the second μ_2_–O^2–^ moiety. The decrease in oxidation potential upon oxide incorporation is consistent with the analogous trend observed between **V**_**6**_**O**_**7**_ and **V**_**6**_**O**_**8**_,^20^ as well as previous reporting from our group, wherein bridging oxide alkylation on a tris-alkoxide functionalized Lindqvist POV-alkoxide, [V_6_O_13_(TRIOL^R^)_2_]^2–^ (R = CH_3_, NO_2_), leads to an anodic shift in cluster redox potentials.^21^

The sensitivity of redox potentials toward surface group identity is similarly demonstrated in the CV of **V**_**6**_**O**_**10**_. The voltammogram of **V**_**6**_**O**_**10**_ features three electrochemical waves (*E*_1/2_ = −0.55, +0.03, and +0.64 V vs Fc^+/0^) with an open circuit voltage at +0.16 V. Thus, the most reduced state accessible to this species features a V_3_^IV^V_3_^V^ oxidation state distribution, demonstrating further restriction of reducibility in comparison to its more ligand-dense congeners. The presence of only one oxidation event with respect to the neutral state is expected, as this would correspond to a diamagnetic, isovalent V_6_^V^ core. Considering the analogous couples in **V**_**6**_**O**_**9**_, the redox potentials of **V**_**6**_**O**_**10**_ are cathodically shifted by ∼0.50 V. The stepwise shift in potentials across this series reveals that the only redox couple shared with **V**_**6**_**O**_**7**_, V_2_^IV^V_4_^V^ + e^–^ ⇌ V_3_^IV^V_3_^V^ is found to be shifted by over −1.3 V relative to the ligand-saturated species, establishing the significant electronic implications of ligand substitution at the surface of the cluster.

Electronic communication between metal centers comprising the cluster cores can be assessed through the determination of comproportionation constants, *K*_c_. This parameter is used to describe the delocalization of electrons in mixed-valent systems by considering the equilibrium state reached upon mixing isostructural species featuring different electron counts (e.g., [V_6_O_7_]^1–^ + [V_6_O_7_]^1+^ ⇌ [V_6_O_7_]).^56,57^ It is calculated from electrochemical data by the following equation (eq 1)
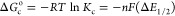
1where Δ*G*_c_^o^ is the free energy of the comproportionation reaction, *R* is the universal gas constant, *T* is the temperature in K, *n* is the number of electrons, *F* is Faraday’s constant, and Δ*E*_1/2_ is the difference between neighboring redox events. For all calculations herein, *T* = 298 K and *n* = 1. In Hartl’s 2009 report on **V**_**6**_**O**_**7**_ and **V**_**6**_**O**_**8**_, it is demonstrated that the installation of the μ_2_–O^2–^ moiety enhances delocalization due to antiferromagnetic super-exchange promoted by the oxide bridge.^20^ This is reflected in the observation that the *K*_c_ values of **V**_**6**_**O**_**8**_ are an order of magnitude greater than those determined for **V**_**6**_**O**_**7**_ (*K*_c_^avg^ = 3.3 × 10^9^ and 8.5 × 10^8^, respectively). Similarly, we find that *K*_c_ increases upon further cluster oxidation, such that **V**_**6**_**O**_**7**_ < **V**_**6**_**O**_**8**_ < **V**_**6**_**O**_**9**_ (*K*_c_^avg^ = 1.0 × 10^10^) < **V**_**6**_**O**_**10**_ (*K*_c_^avg^ = 1.3 × 10^11^). The observed increase of *K*_c_ values with decreased alkoxide density is consistent with Hartl’s results as well as observations from our group on a tris-alkoxide-functionalized cluster with varying degrees of alkylation, [V_6_O_13–*x*_((OCH_2_)_3_CR)_2_(OCH_3_)_*x*_]^(*x*−2)^ (R = CH_3_, NO_2_; *x* = 0, 1, 2).^21^ We note that *K*_c_ between the most reductive features of **V**_**6**_**O**_**10**_ does not agree with this trend (*K*_c_ = 5.0 × 10^8^), suggesting that electronic exchange may be inhibited in more reduced forms of this complex in particular. A similar inconsistency was observed for a triol-bridged cluster bearing a single methoxide moiety, [V_6_O_12_((OCH_2_)_3_CR)_2_(OCH_3_)]^1–^, which features a greater *K*_c_ than its unmethylated analogue, [V_6_O_13_((OCH_2_)_3_CR)_2_]^2–^. Thus, despite the general trends in ligand density/electronic exchange processes, further investigations into the electronic structure of the cores of POV-alkoxides are needed to fully understand delocalization in these complexes (see Table 3).

**Table 3:** Comproportionation Equilibrium Constants, *K*_c_ (298 K), Calculated from the Redox Potentials of Each Complex

	charge states			
compound	2–/0	1–/1+	0/2+	average
**V**_**6**_**O**_**7**_	2.9 × 10^8^	9.2 × 10^8^	1.4 × 10^9^	8.5 × 10^8^
**V**_**6**_**O**_**8**_	1.4 × 10^9^	2.0 × 10^9^	6.5 × 10^9^	3.3 × 10^9^
**V**_**6**_**O**_**9**_	1.1 × 10^10^	9.5 × 10^9^	1.0 × 10^10^	1.0 × 10^10^
**V**_**6**_**O**_**10**_	5.0 × 10^8^	2.6 × 10^11^		1.3 × 10^11^

## Conclusions

Capping ligands influence the solubility, reactivity, and electronics of nanoparticles. However, the challenges associated with synthesizing and studying these materials with atomic precision make controlling the ligand density on a material surface and understanding the role(s) of surface coverage important hurdles to cross in material development. To provide insights into the influence of ligand density, we present a new route to the isolation of POV-alkoxide clusters featuring μ_2_–O^2–^ ligands via post-synthetic modification of Lindqvist-type POV-alkoxide clusters. [V_6_O_7_(OCH_3_)_12_]^1+^ (**V**_**6**_**O**_**7**_^**1+**^) is converted to V_6_O_8_(OCH_3_)_11_ (**V**_**6**_**O**_**8**_) and V_6_O_9_(OCH_3_)_10_ (**V**_**6**_**O**_**9**_) when exposed to water. Subsequent installation of μ_2_–O^2–^ ligands is possible via addition of water to [V_6_O_8_(OCH_3_)_11_]^1+^, resulting in the formation of V_6_O_10_(OCH_3_)_9_ (**V**_**6**_**O**_**10**_). The Lindqvist core structure is retained across all four derivatives, with changes in bond metrics required to tolerate the inclusion of bridging oxides. These structural alterations have implications on the electronic structure of the cluster series, most notably in the substantial shift in redox potentials observed upon each subsequent ligand exchange (Δ*E*_1/2,avg_ = −0.45 V). Additionally, the degree of cluster reduction is restricted upon installation of bridging oxides, with the dodecamethoxide species **V**_**6**_**O**_**7**_ able to access a V_6_^IV^ core and its most oxidized congener **V**_**6**_**O**_**10**_ only able to be reduced to the V_3_^V^V_3_^IV^ state. Finally, the delocalization of electrons throughout the vanadium oxide cores is demonstrably enhanced upon oxidation, with each μ_2_–O^2–^ moiety eliciting an increase in the comproportionation constants by an order of magnitude. These results indicate how surface ligands may influence the physicochemical properties of nanoscopic metal oxides.

This work highlights a few peculiarities of the POV-alkoxide cluster system studied by our team. POV-methoxide clusters react with water only in their cationic charge state, revealing the oxidative instability of the Lindqvist core capped with methoxide ligands. Additionally, reactivity with water is only observed in the case of the methoxide-capped derivative; bridging ethoxide ligands at the surface of the assembly stabilize the hexavanadate core in cationic charge states, limiting ligand exchange reactions under the investigated conditions.

The results presented here have posed a number of exciting avenues of research, all of which are under active investigation by our team. Most significantly, the exposed μ_2_–O^2–^ surface moieties provide nucleophilic reactive sites, making these complexes intriguing candidates for electrochemical water oxidation and other multielectron/multiproton reactivity.

## Supporting Information Available

Crystallographic parameters for **V**_**6**_**O**_**9**_ and **V**_**6**_**O**_**10**_, ^1^H NMR spectra, ESI–MS data, and IR spectra of all complexes reported (PDF)
