# Supplementary material for: Manipulating Ligand Density at the Surface of Polyoxovanadate-Alkoxide Clusters
Source: Inorg Chem. 2023 Sep 15;62(38):15616–26. doi: 10.1021/acs.inorgchem.3c02314 (PMC10523436; doi:10.1021/acs.inorgchem.3c02314)
Supplement: Supplementary file 1 — ic3c02314_si_001.pdf [file ic3c02314_si_001.pdf]

Electronic Supporting Information (ESI)

Manipulating Ligand Density at the Surface of Polyoxovanadate-Alkoxide Clusters

Thompson V. Marinho,[a]‡ Eric Schreiber,[a]‡ Rachel E. Garwick, William W. Brennessel,[a] and Ellen M. Matson[a]*

[a]Department of Chemistry, University of Rochester, Rochester, New York 14627

‡ Authors contributed equally to this work

*Corresponding author email: matson@chem.rochester.edu

Supporting Information Table of Contents

Table S1. Crystallographic parameters for molecular structures of $\mathbf{V_6O_9}$ and $\mathbf{V_6O_{10}}$. S2

Figure S1. ^{1}H NMR spectrum of crude reaction of $\mathbf{V_6O_7{}^{1+}}$ and D_2O S3

Figure S2. Time elapsed ^{1}H NMR spectra of crude reaction of $\mathbf{V_6O_7}$ and 150 equiv H_2O........................S3

Figure S3. Time elapsed ^{1}H NMR spectra of crude reaction of $\mathbf{V_6O_7{}^{1-}}$ and 150 equiv H_2O.....................S4

Figure S4. Time elapsed ^{1}H NMR spectra of crude reaction of $\mathbf{V_6O_7{}^{2-}}$ and 150 equiv H_2O.....................S4

Figure S5. ESI-MS of crude reaction of $\mathbf{V_6O_7{}^{1+}}$ and H_2O S5

Figure S6. Cyclic voltammogram of crude reaction of $\mathbf{V_6O_7{}^{1+}}$ and H_2O S5

Figure S7. Infrared spectra of $\mathbf{V_6O_7}$, $\mathbf{V_6O_8}$, $\mathbf{V_6O_9}$, and $\mathbf{V_6O_{10}}$ S6

Figure S8. ESI-MS(-)ve of eluent containing $\mathbf{V_6O_9}$ S6

Figure S9. ^{1}H NMR spectrum of $\mathbf{V_6O_9}$ S7

Table S2. Bond valence sum calculations for $\mathbf{V_6O_9}$. S7

Figure S10. ^{1}H NMR spectrum of crude reaction of $\mathbf{[V_6O_7(OC_2H_5)_{12}]^{1+}}$ and H_2O S7

Figure S11. ^{1}H NMR spectrum of crude reaction of $\mathbf{[V_6O_7(OC_2H_5)_{12}]^{2+}}$ and H_2O S8

Figure S12. ^{1}H NMR spectrum of $\mathbf{V_6O_8{}^{1+}}$...S8

Figure S13. Infrared spectra of $\mathbf{V_6O_8}$ and $\mathbf{V_6O_8{}^{1+}}$...S9

Table S3. Bond valence sum calculations for $\mathbf{V_6O_{10}}$. S9

Figure S14. ESI-MS of crude reaction of $\mathbf{V_6O_8{}^{1+}}$ and H_2O……………………………………………….…S10

Figure S15. ^{1}H NMR spectrum of $\mathbf{V_6O_{10}}$……………………………………………………………………….S11

References. ………………………………...……………………………………………………………….S11

Table S1. Crystallographic parameters for molecular structures of **V_6O_9** and **V_6O_{10}**.

Compound	**V_6O_9**	**V_6O_{10}**
Empirical formula	$C_{10}H_{30}O_{19}V_6$	$C_9H_{27}O_{19}V_6$
Formula weight	759.98	744.94
Temperature / K	99.98(10)	100.00(10)
Wavelength / Å	1.54184	1.54184
Crystal group	Monoclinic	Monoclinic
Space group	$P2_1/c$	$P2_1$/n
Unit cell dimensions	*a* =15.1976(2) Å<br>*b* =9.24888(140 Å<br>*c* =17.6701(2) Å<br>*α* = 90°<br>*β* = 93.4587(13)°<br>*γ* = 90°	*a* = 9.87550(10) Å<br>*b* = 15.4732(2) Å<br>*c* = 15.7062(2) Å<br>*α* = 90°<br>*β* = 96.4070(10)°<br>*γ* = 90°
Volume / $Å^3$	2479.20(6)	2385.01(5)
Z	4	4
Reflections collected	9420	41110
Independent reflections	8668	4418
Completeness (theta)	99.9% (74.504°)	100.0% (67.684°)
Goodness-of-fit on F^2	1.093	1.106
Final *R* indices [*I*>2sigma(*I*)]	*R*1 = 0.0595	*R*1 = 0.0399
Largest diff. peak and hole	1.704 and -1.305 e.$Å^{-3}$	0.552 and -0.718 e.$Å^{-3}$

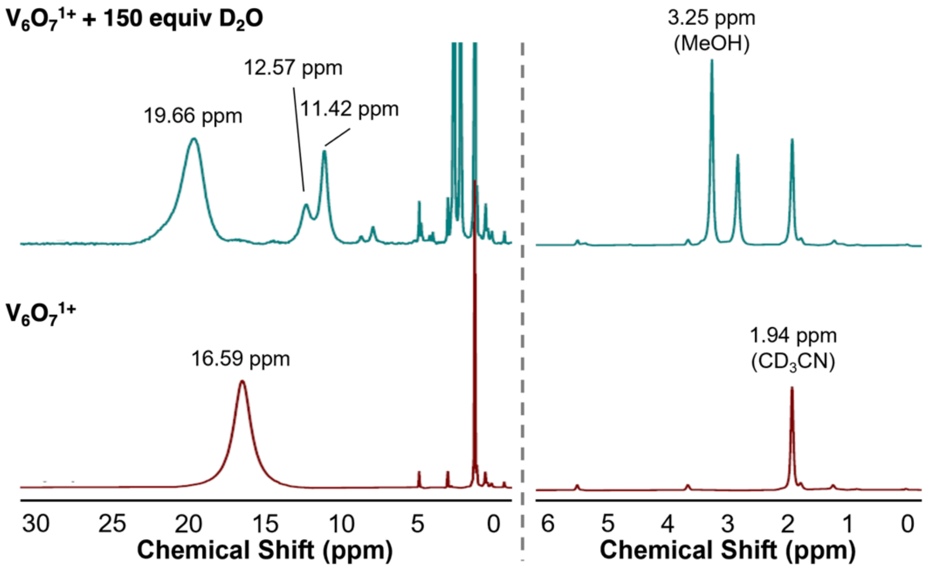

Figure S1. ^{1}H NMR spectrum of the crude reaction performed in a J-Young tube of **$V_6O_7$$^{1+}$** and 150 equiv of D_2O collected in CD_3CN at 21 °C after 30 min; inset shows formation of methanol following addition of D_2O (top). [1]H NMR spectrum of the starting material, **$V_6O_7$$^{1+}$**, collected in CD_3CN at 21 °C (bottom).

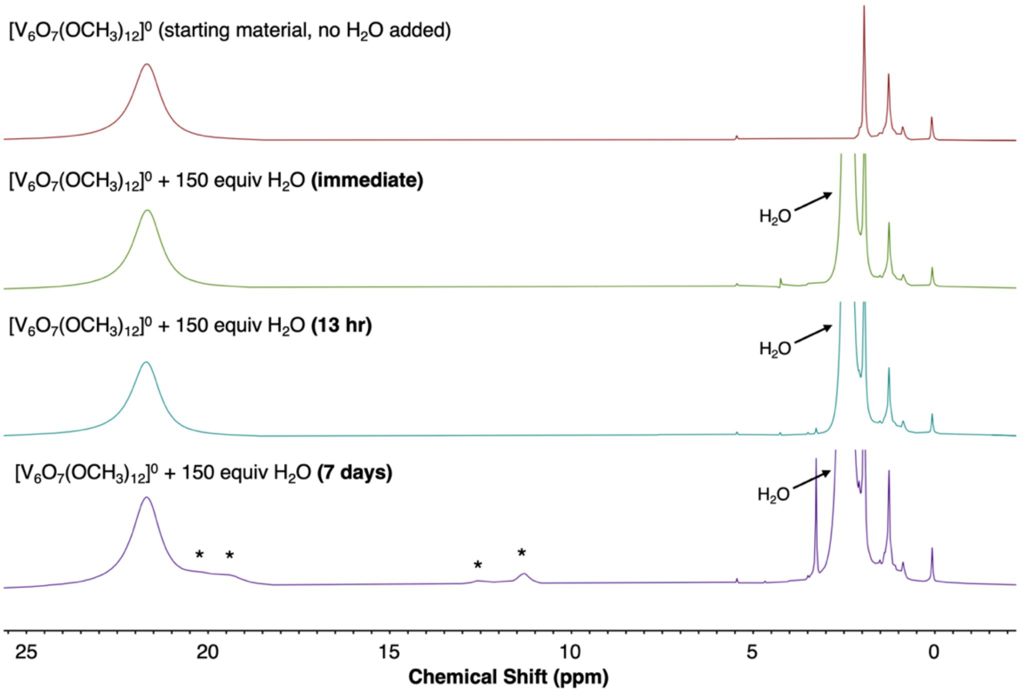

Figure S2. ^{1}H NMR spectra of crude reaction of **$[V_6O_7(OCH_3)_{12}]^0$** and 150 equiv H_2O. Time points taken immediately after addition (green), 13 hours after addition of water (teal), and one week after addition of water (purple, bottom). (*) signals indicate formation of V_6O_8 in the bottom spectrum, suggesting conversion of the neutral POV-alkoxide cluster to V_6O_8 is possible after prolonged exposure to water.

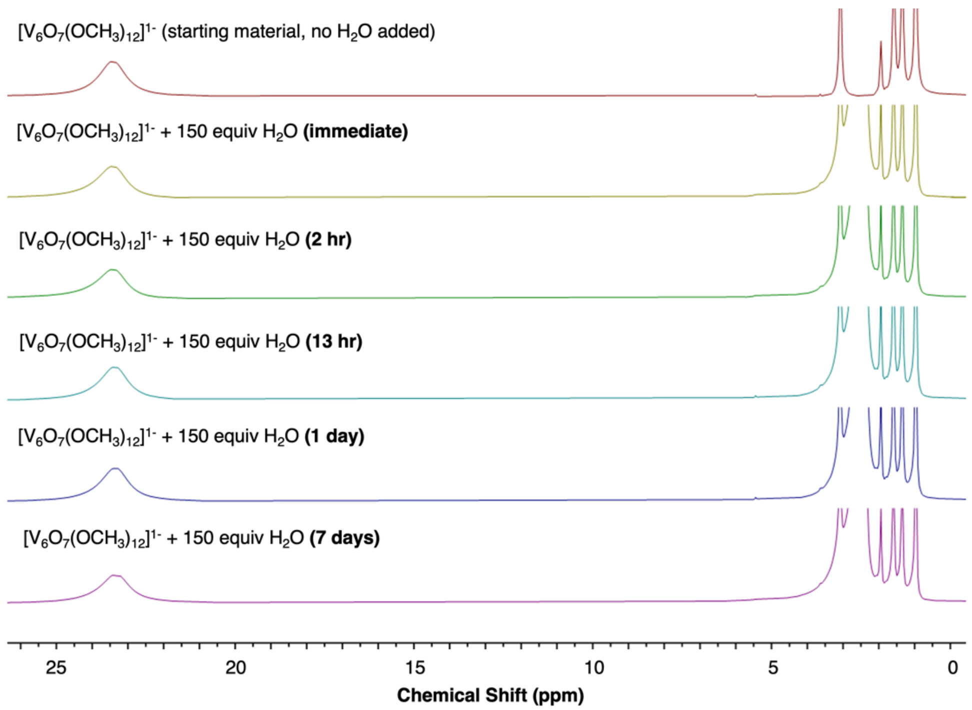

Figure S3. ^{1}H NMR spectra of crude reaction of **$[V_6O_7(OCH_3)_{12}]^{1-}$** and 150 equiv H_2O. Top spectrum is that of the starting material, $[V_6O_7(OCH_3)_{12}]^{1-}$ in the absence of water (red, top). Time points taken immediately after addition (yellow-green), 2 hours after addition of water (light-green), 13 hours after addition (green), and 1 day after addition (blue), and 7 days after addition of water (purple, bottom).

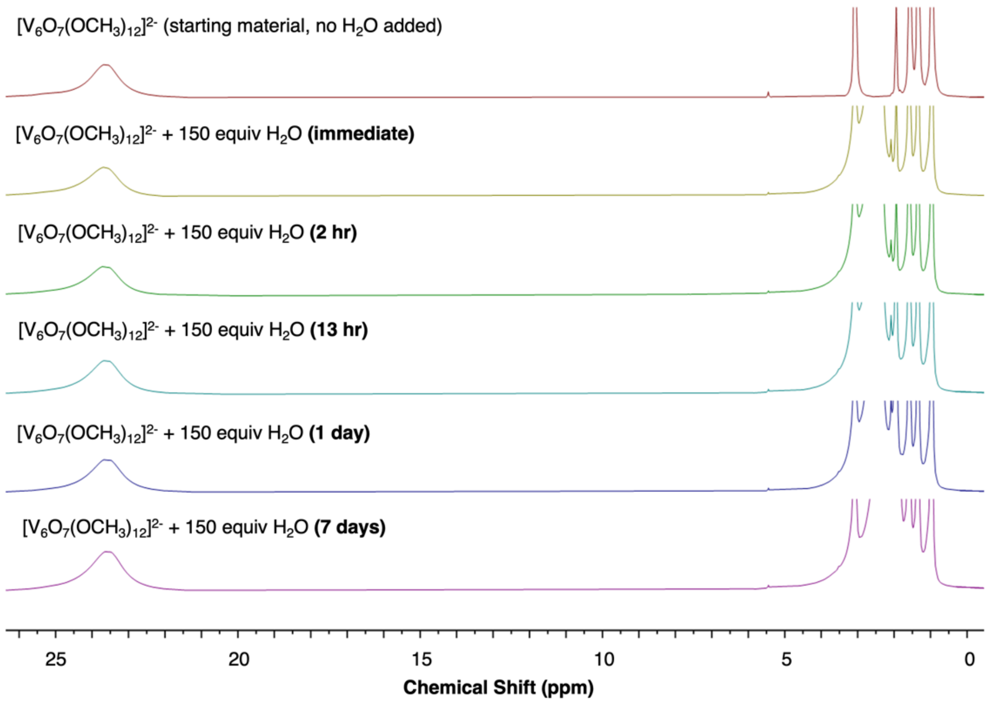

Figure S4. ^{1}H NMR spectra of crude reaction of **$[V_6O_7(OCH_3)_{12}]^{2-}$** and 150 equiv H_2O. Top spectrum is that of the starting material, $[V_6O_7(OCH_3)_{12}]^{2-}$ in the absence of water (red, top). Time points taken immediately after addition (yellow-green), 2 hours after addition of water (light-green), 13 hours after addition (green), and 1 day after addition (blue), and 7 days after addition of water (purple, bottom).

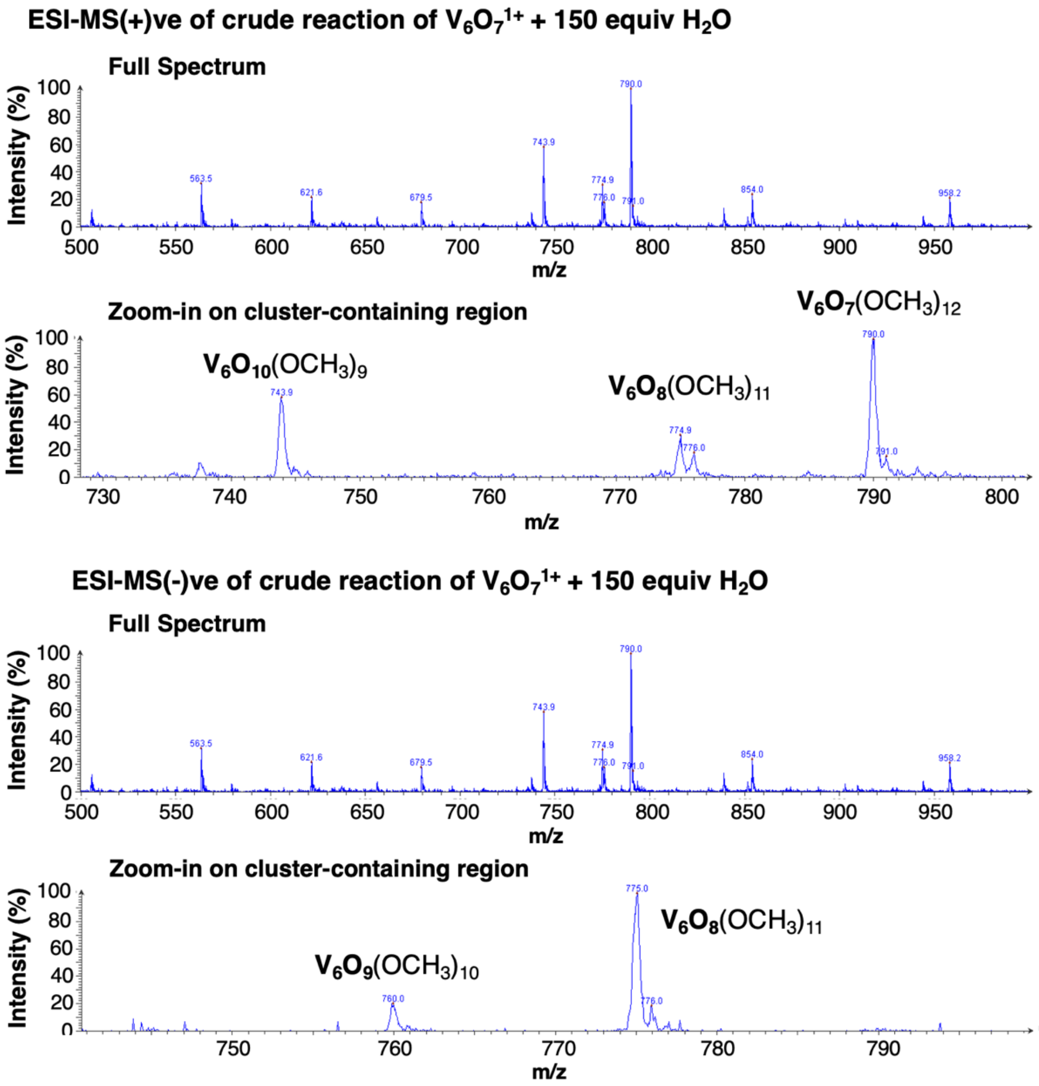

Figure S5. ESI-MS data for the crude reaction of **[$V_6O_7(OCH_3)_{12}$]$^{1+}$** and 150 equiv H_2O.

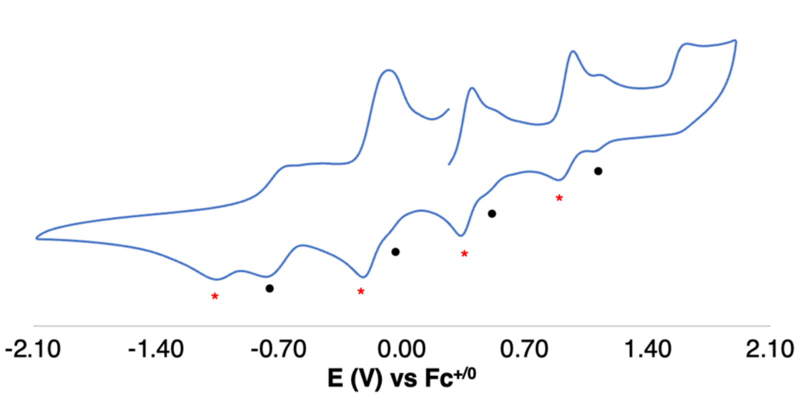

Figure S6. Cyclic voltammogram of crude reaction of **$V_6O_7$$^{1+}$** and H_2O collected in acetonitrile with 0.1 M [$^nBu_4N$][PF_6] as the supporting electrolyte (100 mv/s). (*) indicate peaks that correspond to **V_6O_8** and (•) indicate peaks that correspond to **V_6O_9**.[1]

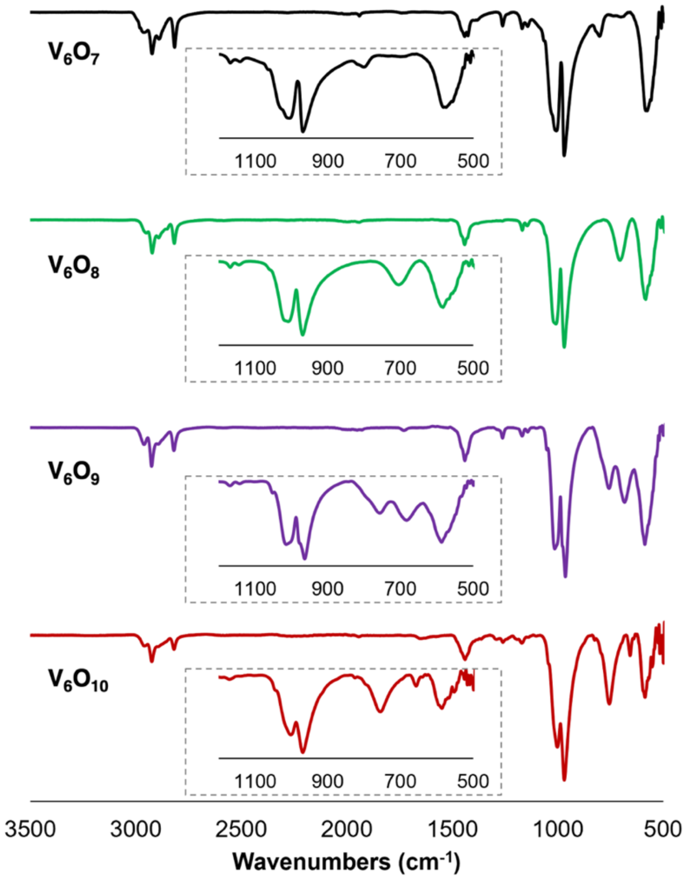

Figure S7. From top to bottom, infrared spectra of **V_6O_7** (black), **V_6O_8** (green), **V_6O_9** (purple), and **V_6O_{10}** (red). Inset shows a magnification of the region between 500 and 1100 cm^{-1} where pertinent bands of the cluster core are observed.

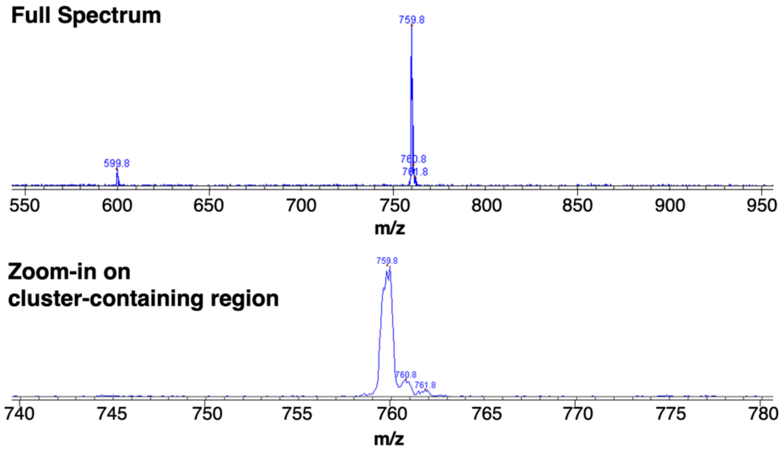

Figure S8. ESI-MS(-)ve of column eluent containing **V_6O_9** (m/z = 760).

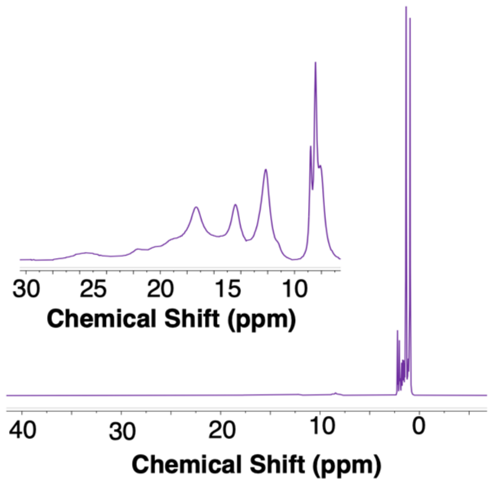

Figure S9. ^{1}H NMR spectrum of a crystalline sample of $\mathbf{V_6O_9}$ collected in CD_3CN at 21°C.

Table S2. Bond valence sum calculations for $\mathbf{V_6O_9}$ based on X-Ray crystallographic data collected at 100 K. Table reflects the results of BVS calculations using V-O valence parameters (r_0) for different oxidation states of vanadium.

$\mathbf{V_6O_9^0}$	V1	V2	V3	V4	V5	V6
V(III)	4.606	4.592	4.067	4.475	3.956	4.587
V(IV)	4.716	4.701	**4.164**	4.582	**4.050**	4.697
V(V)	**5.016**	**5.001**	4.436	**4.876**	4.318	**4.999**

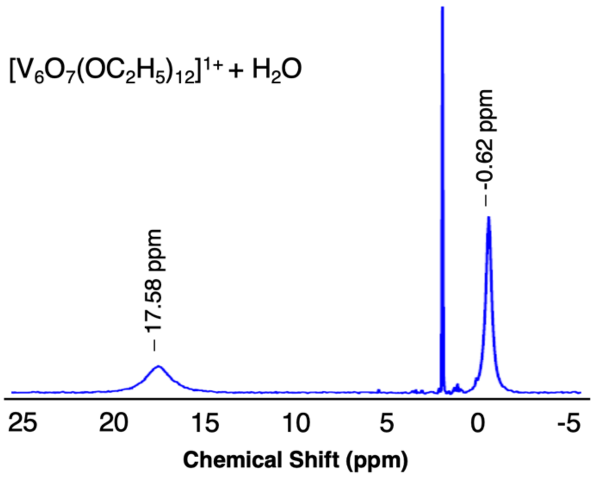

Figure S10. ^{1}H NMR spectrum of $\mathbf{[V_6O_7(OC_2H_5)_{12}]^{1+}}$ with H_2O added collected in CD_3CN at 21 °C. Spectrum is consistent with peaks of the starting material.[2, 3]

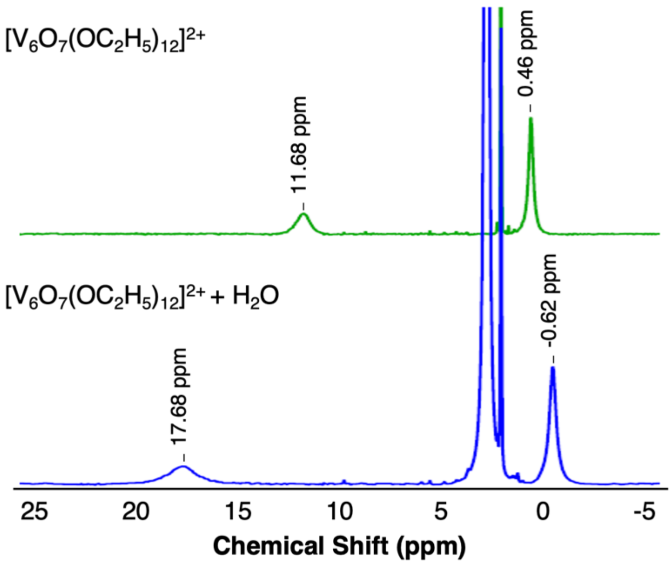

Figure S11. ^{1}H NMR spectrum of **[$V_6O_7(OC_2H_5)_{12}]^{2+}$ (**top, green) and **[$V_6O_7(OC_2H_5)_{12}]^{2+}$** (bottom, blue) **with** H_2O added collected in CD_3CN at 21 °C. Bottom spectrum shows conversion of [$V_6O_7(OC_2H_5)_{12}]^{2+}$ to [$V_6O_7(OC_2H_5)_{12}]^{1+}$ in the presence of H_2O.[2, 3]

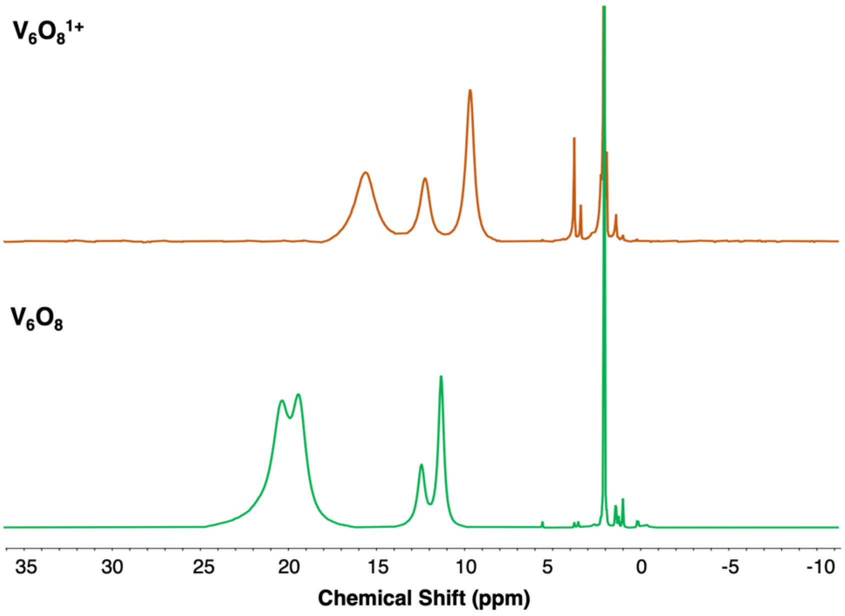

Figure S12. ^{1}H NMR spectrum of **$V_6O_8^{1+}$** (top, orange), stacked against the neutral cluster, **V_6O_8** (bottom, green). Spectra are collected in CD_3CN at 21°C.

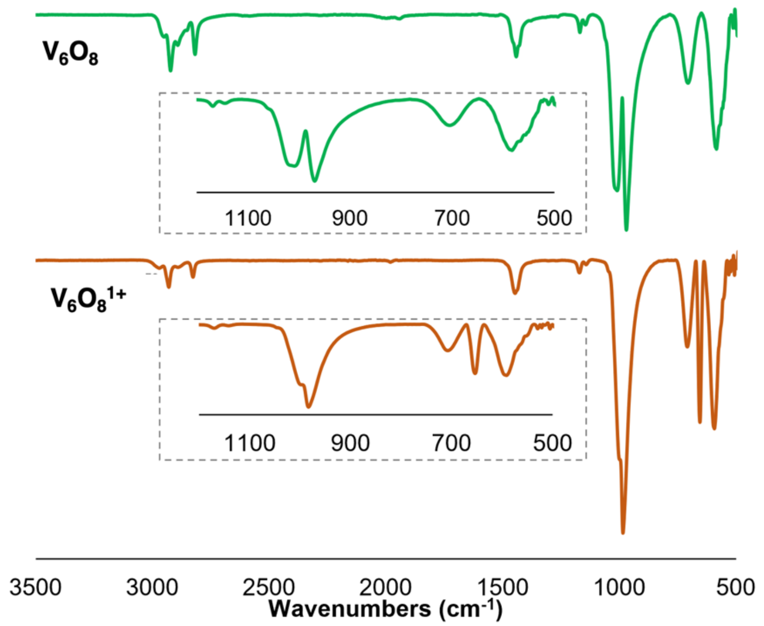

Figure S13. Infrared spectra of **V_6O_8** (top, green) and **$V_6O_8^{1+}$** (bottom, orange). Inset shows a magnification of the region between 500 and 1100 cm^{-1} where pertinent bands of the cluster core are observed.

Table S3. Bond valence sum calculations for **V_6O_{10}** based on X-Ray crystallographic data collected at 100 K. Table reflects the results of BVS calculations using V-O valence parameters (r_0) for different oxidation states of vanadium.

**$V_6O_{10}^0$**	V1	V2	V3	V4	V5	V6
V(III)	4.654	4.539	4.625	3.994	4.542	4.589
V(IV)	4.765	4.647	4.735	**4.089**	4.651	4.699
V(V)	**5.066**	**4.942**	**5.035**	4.359	**4.951**	**5.000**

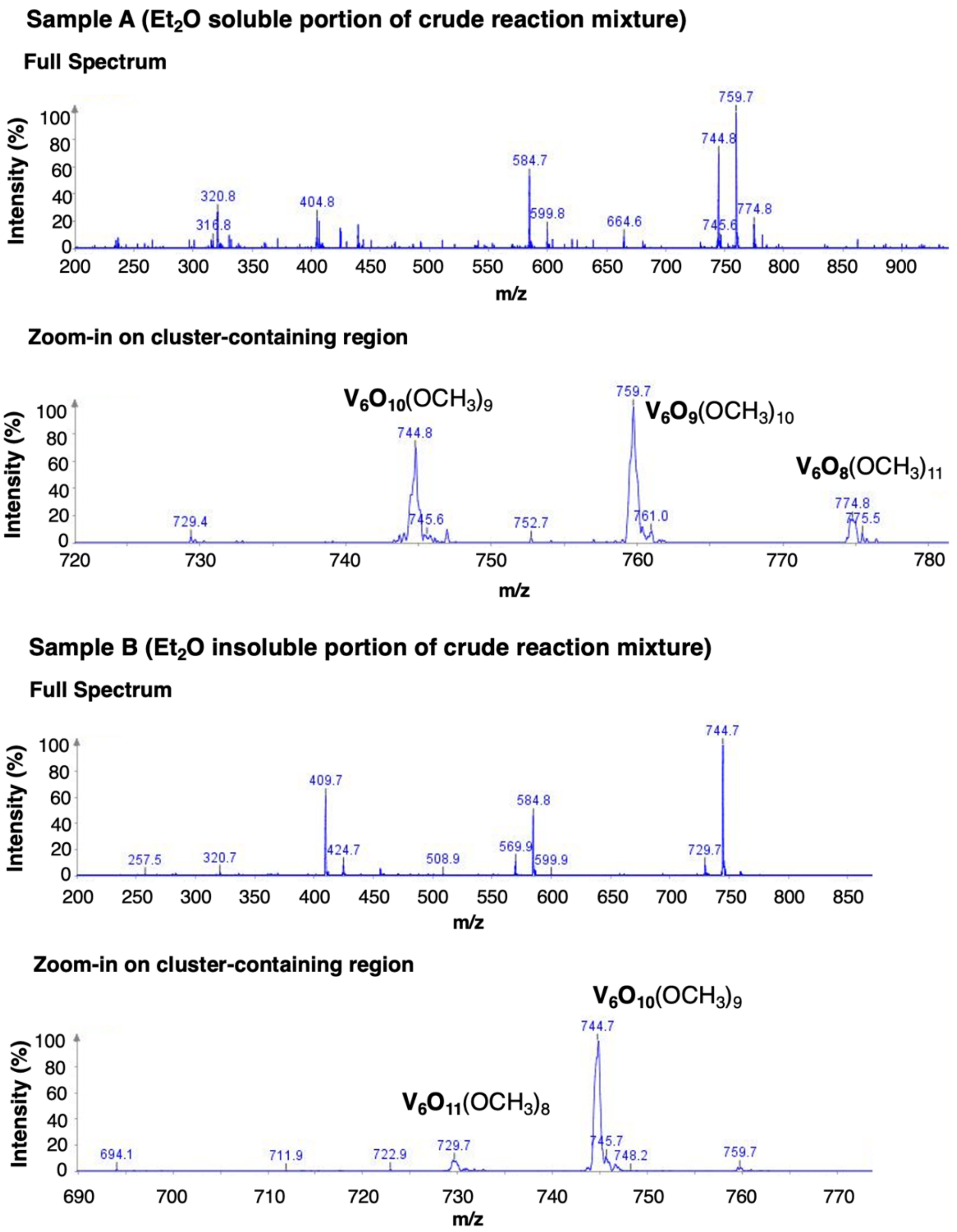

Figure S14. ESI-MS(-)ve of reaction of $\mathbf{V_6O_8}^{1+}$ and H_2O; Sample A contains portion of product that is *soluble* in Et_2O (top, both full spectrum and cluster-containing region shown for clarity), while Sample B contains portion of product that is *insoluble* in Et_2O (bottom, both full spectrum and cluster-containing region are shown for clarity). As evidenced from this data, a mixture of products, $\mathbf{V_6O_9}$ **(minor)**, $\mathbf{V_6O_{10}}$, and $\mathbf{V_6O_{11}}$ **(minor),** is formed. Only $\mathbf{V_6O_{10}}$ could be isolated in meaningful yield.

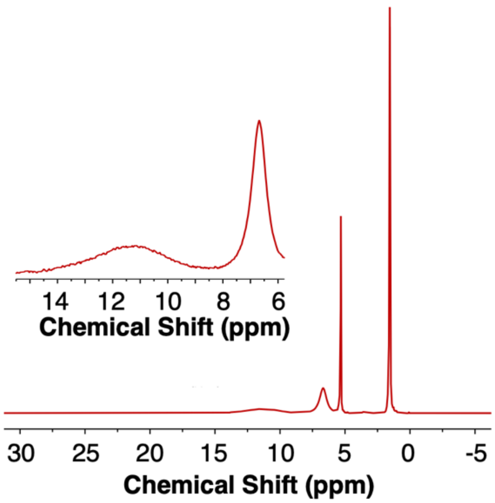

Figure S15. ^{1}H NMR spectrum of $\mathbf{V_6O_{10}}$ collected in CD_2Cl_2 at 21°C.

References.

(1) Daniel, C.; Hartl, H. A Mixed-Valence V^{IV}/V^{V} Alkoxo-polyoxovanadium Cluster Series $[V_6O_8(OCH_3)_{11}]^{n+/-}$: Exploring the Influence of a μ-Oxo Ligand in a Spin Frustrated Structure. *Journal of the American Chemical Society* **2009**, *131*, 5101-5114.

(2) Daniel, C.; Hartl, H. Neutral and Cationic VIV/VV Mixed-Valence Alkoxo-polyoxovanadium Clusters $[V_6O_7(OR)_{12}]^{n+}$ (R = $-CH_3$, $-C_2H_5$): Structural, Cyclovoltammetric and IR-Spectroscopic Investigations on Mixed Valency in a Hexanuclear Core. *Journal of the American Chemical Society* **2005**, *127*, 13978-13987.

(3) VanGelder, L. E.; Kosswattaarachchi, A. M.; Forrestel, P. L.; Cook, T. R.; Matson, E. M. Polyoxovanadate-alkoxide clusters as multi-electron charge carriers for symmetric non-aqueous redox flow batteries. *Chemical Science* **2018**, *9*, 1692-1699.
